# Human iPSC-Derived Neurons with Reliable Synapses and Large Presynaptic Action Potentials

**DOI:** 10.1523/JNEUROSCI.0971-23.2024

**Published:** 2024-05-09

**Authors:** Torsten Bullmann, Thomas Kaas, Andreas Ritzau-Jost, Anne Wöhner, Toni Kirmann, Filiz Sila Rizalar, Max Holzer, Jana Nerlich, Dmytro Puchkov, Christian Geis, Jens Eilers, Robert J. Kittel, Thomas Arendt, Volker Haucke, Stefan Hallermann

**Affiliations:** ^1^Carl-Ludwig-Institute of Physiology, Faculty of Medicine, Leipzig University, Leipzig 04103, Germany; ^2^Leibniz-Forschungsinstitut für Molekulare Pharmakologie (FMP), Berlin 13125, Germany; ^3^Paul-Flechsig-Institute for Brain Research, Faculty of Medicine, Leipzig University, Leipzig 04103, Germany; ^4^Section Translational Neuroimmunology, Department of Neurology, Jena University Hospital, Jena 07747, Germany; ^5^Institute of Biology, Department of Animal Physiology, Leipzig University, Leipzig 04103, Germany; ^6^Faculty of Biology, Chemistry, Pharmacy, Freie Universität Berlin, Berlin 14195, Germany

**Keywords:** action potential, human, iPSC, presynaptic

## Abstract

Understanding the function of the human brain requires determining basic properties of synaptic transmission in human neurons. One of the most fundamental parameters controlling neurotransmitter release is the presynaptic action potential, but its amplitude and duration remain controversial. Presynaptic action potentials have so far been measured with high temporal resolution only in a limited number of vertebrate but not in human neurons. To uncover properties of human presynaptic action potentials, we exploited recently developed tools to generate human glutamatergic neurons by transient expression of Neurogenin 2 (Ngn2) in pluripotent stem cells. During maturation for 3 to 9 weeks of culturing in different established media, the proportion of cells with multiple axon initial segments decreased, while the amount of axonal tau protein and neuronal excitability increased. Super-resolution microscopy revealed the alignment of the pre- and postsynaptic proteins, Bassoon and Homer. Synaptic transmission was surprisingly reliable at frequencies of 20, 50, and 100 Hz. The synchronicity of synaptic transmission during high-frequency transmission increased during 9 weeks of neuronal maturation. To analyze the mechanisms of synchronous high-frequency glutamate release, we developed direct presynaptic patch-clamp recordings from human neurons. The presynaptic action potentials had large overshoots to ∼25 mV and short durations of ∼0.5 ms. Our findings show that Ngn2-induced neurons represent an elegant model system allowing for functional, structural, and molecular analyses of glutamatergic synaptic transmission with high spatiotemporal resolution in human neurons. Furthermore, our data predict that glutamatergic transmission is mediated by large and rapid presynaptic action potentials in the human brain.

## Significance Statement

Presynaptic physiology remains poorly understood despite its relevance to neurological and psychiatric diseases. Human induced pluripotent stem cell (iPSC) derived neurons are a convenient tool to study the physiological and pathophysiological mechanisms of neurotransmitter release in human neurons. As a first step, we established direct presynaptic whole-cell patch-clamp recordings from human glutamatergic neurons induced by transient Neurogenin 2 overexpression. We furthermore analyzed the structure of the synapses with super-resolution light microscopy and the synaptic short-term plasticity during high-frequency transmission. Our findings show that synchronous high-frequency transmission is mediated by rapid and large presynaptic action potentials in human neurons, similar to small conventional nerve terminals of rodent neurons.

## Introduction

Neuronal communication relies on precisely timed neurotransmitter release at presynaptic nerve terminals (Südhof, 2012). Despite intense efforts, several fundamental questions regarding presynaptic function and plasticity remain unresolved (Neher and Sakaba, 2008; Debanne et al., 2011; Südhof, 2013; Kaeser and Regehr, 2014). Further research is needed because presynaptic function and plasticity play important roles in learning, memory, and the development of neurological and psychiatric diseases (Hawkins et al., 1993; Monday et al., 2018; Verhage and Sørensen, 2020). The presynaptic mechanisms are particularly poorly understood in human neurons. Furthermore, recent studies have suggested fundamental differences in the biophysical properties, the function, and the morphology of human neurons compared with the widely studied neurons of mice and rats (Eyal et al., 2016; Beaulieu-Laroche et al., 2018; Fişek and Häusser, 2020; Gidon et al., 2020). Animal models often fail to recapitulate human brain disease phenotypes or to predict treatment outcomes (Miller, 2010). Recently available induced pluripotent stem cell (iPSC)-derived neurons therefore hold promise for a better understanding of presynaptic function in human neurons in health and disease. In addition, iPSC-derived human neurons have the potential to replace some of the animal experiments in biomedical research representing an efficient 3R strategy (Russell and Burch, 1959).

To study synaptic mechanisms in human neurons, several approaches have been used, including acute slices from surgically resected brain tissue as well as organoid and cell cultures derived from iPSCs or fibroblasts. Each of these model systems has complementary advantages and disadvantages. Ex vivo brain tissue best represents the morphological and functional organization of the adult human brain (Molnár et al., 2016; Hodge et al., 2019; Peng et al., 2024). However, brain tissue from patients is limited in its availability, the physiology might be compromised by the pathology of patients, and genetic manipulations are complicated (Ting et al., 2018). A more accessible source are human fibroblasts and iPSCs that can be converted into neurons. The most common model system are cultured neurons derived from fibroblasts or iPSCs by pharmacological treatments or transient expression of proneural transcription factors.

An important milestone has been the finding that transient expression of the transcription factor Ngn2 suffices to rapidly induce the development of glutamatergic neurons from human iPSCs (Zhang et al., 2013). Ngn2 overexpression has been proven as a valuable tool to produce glutamatergic human neurons (Hulme et al., 2022). By now, there are several human iPSC lines with endogenous inducible Ngn2 available, including some cell lines with mutations linked to neurodegenerative diseases (Ghatak et al., 2019; Karch et al., 2019; Nakamura et al., 2019; Sohn et al., 2019; Tracy et al., 2022; Zhou et al., 2022). Human neurons produced by Ngn2 expression exhibit markers of pyramidal neurons of the neocortex (Zhang et al., 2013) but several types of neurons might be present depending on the exact culture condition and the Ngn2 dosage (Lin et al., 2021). Recently, it was shown that human neurons induced by both pharmacological treatment and overexpressing Ngn2 can be cultured in islands to form “autapses” with reproducible neuronal and synaptic properties (Fenske et al., 2019; Meijer et al., 2019; Rhee et al., 2019).

Here, we generated cultured human neurons derived from iPSCs by transient forced expression of Ngn2 (Zhang et al., 2013; Patzke et al., 2019). In general, cultured neurons offer ideal accessibility for high-resolution presynaptic electrophysiology (Kawaguchi and Sakaba, 2017; Ritzau-Jost et al., 2023) and for super-resolution imaging (Werner et al., 2021). Therefore, we compared various culturing media and analyzed the structural, molecular, and functional maturation of iPSC-derived human neurons. Furthermore, we analyzed glutamatergic synaptic transmission. We found high-fidelity synaptic transmission with synchronous release up to 100 Hz mediated by large and rapid presynaptic action potentials.

## Material and Methods

### Laboratory animals

Cortical astrocytes were prepared using C57BL/6N mice of either sex at postnatal day (P) 0–3. Mixed cultures of rat and mouse cortical neurons and astrocytes were prepared using rat of either sex at P1. Adult animals were kept in individually ventilated cages and received water and food *ad libitum*. All animal procedures were in accordance with the European (EU Directive 2010/63/EU, Annex IV for animal experiments), national, and Leipzig University guidelines. All animal procedures were approved in advance by the institutional Leipzig University Ethics Committees and the federal Saxonian Animal Welfare Committee (mouse, T01/21; rat, T29/21). In order to reduce the number of animals, we tried to use remaining cells from other neuron culture preparations in our laboratory whenever possible.

### Human-induced neuron (hiN) culture

Human-induced pluripotent stem cells (hiPSCs) were differentiated into glutamatergic neurons of cortical subtype by the transient overexpression of the transcription factor Neurogenin 2 (Ngn2; Zhang et al., 2013). The hiPSC line BIHi005-A-24 (hPSCreg link: https://hpscreg.eu/cell-line/BIHi005-A-24) stably overexpressing the TetO-NGN2-T2A-PURO cassette was obtained from Berlin Institute of Health (BIH), Germany. This cell line constitutively expresses a Tet-ON activator which in turn allows induction of Ngn2 expression and the puromycin resistance gene by addition of doxycycline to the culture medium (Rizalar et al., 2023). For capacitance measurements, we used the iPSC line NWTC11.G3.0036 (Karch et al., 2019) derived from WTC11 (hPSCreg link: https://hpscreg.eu/cell-line/UCSFi001-A), which also constitutively expresses a Tet-ON activator and was differentiated into glutamatergic neurons using the same protocol. Since the morphological apparency of the neurons and the functional essays did not reveal any differences between the two cell lines, we expect similar fundamental biophysical properties of presynaptic boutons in these cells. In both cell lines, HIV, HBV, HCV, and mycoplasma were not detected by PCR.

Prior differentiation hiPSCs were maintained in feeder-free culture. They were grown on plastic dishes coated with Matrigel (Corning). Medium was replaced every second day by fresh StemFlex Medium (StemCell Technologies) without antibiotics. Upon reaching 50–70%, confluence cells were harvested with StemPro Accutase (Thermo Fisher Scientific) and split. With each passage, StemFlex medium was supplemented with 5 µM ROCK inhibitor Y-27632 (StemCell Technologies) to inhibit apoptosis. Frozen stocks were prepared in Bambanker HRM cryopreservation media (StemCell Technologies) and stored over liquid nitrogen.

For differentiation into neurons, hiPSC cultures where grown until reaching 50–70% confluence cells before being harvested with StemPro Accutase and plated onto Matrigel-coated 6-well plates at 200,000 cells/well in 2 ml/well StemFlex medium supplemented with 5 µM ROCK inhibitor Y-27632 to inhibit apoptosis. The following 3 d (Day 0–2), the medium was replaced daily with 2 ml/well fresh differentiation medium (DMEM/F12, 1:100 N2 supplement, 1:100 nonessential amino acids, 1:100 penicillin/streptomycin, all ThermoFisher; 10 ng/ml BDNF, 10 ng/ml NT3, both from StemCell Technologies; 200 ng/ml laminin, 1 µg/ml doxycycline, both from ThermoFisher). Puromycin selection was started by adding 10 mg/ml puromycin (ThermoFisher) on Day 2. hiPSCs rapidly changed morphology toward a bipolar shape with elongated neurites.

Because mouse astrocytes efficiently stimulate synaptogenesis in human-induced neurons (Zhang et al., 2013), cultures from P1 to P3 mice were prepared 2 to 3 weeks earlier with trypsin digestion followed by mechanical dissociation and maintained in astrocyte medium (DMEM with 4.5 g/l glucose, 1 mM pyruvate, 10% heat inactivated fetal calf serum, 1:100 penicillin/streptomycin). They were replated two times to remove any mouse neurons.

On Day 3, neuron cultures were washed with Hanks balanced salt solution (HBSS) to remove puromycin and harvested with Accutase. In some cases frozen stocks were prepared (Ishizuka and Bramham, 2020) and stored at −80°C before use. Astrocyte cultures were harvested with trypsin/EDTA. Cocultures were then plated onto poly-D-lysine and Matrigel-coated coverslips in 24-well plates. For each coverslip, 60,000 neurons and 12,000 astrocytes were allowed to settle in a 25 µl drop of neuron culture medium for ∼30 min and then each well was filled with 500 µl culture medium (Table 1) supplemented with 2% heat-inactivated fetal calf serum (ThermoFisher). Half of the medium was replaced with culture medium on Days 6, 10, and 14 and then twice a week. Doxycycline was added to the medium until Day 10. Cytosine arabinoside (ara-C) was added at Day 12 for 48 h to inhibit overgrowth of astrocytes. Due to osmolarity of the culture media (Table 2) and possible evaporation, changing half of the media led to measurable differences in osmolarity. To compensate for these differences, we adjusted the osmolarity to 300 mOsm using *Aqua dest.* for DMEM, BrainPhys, and Neurobasal using the following approach. Half of the supernatant (250 µl/well) was collected from the cell cultures. The pooled supernatant was then mixed with equal volume of the fresh medium. The volume (*V*_mix_) and the osmolarity (*O*_mix_) of the mixture were measured. The osmolarity was then adjusted by adding a volume of *Aqua dest.* (*V*_add_) according to the following formula: *V*_add _= (*O*_mix_ / 300 mOsm−1) × *V*_mix_. The remaining supernatant was removed from the cell cultures and replaced by the mixture (500 µl/well). For Neurobasal Plus medium, this approach was not possible because the measured osmolarity was always below 300 mOsm (compare Table 2). We therefore simply exchanged half of the Neurobasal Plus medium with fresh medium. Cultures were maintained for up to 10 weeks at 37°C and 5% CO_2_. Concentration of electrolytes as well as glucose and lactate in all culture media as well as in artificial cerebrospinal fluid (ACSF) were measured using a blood gas and electrolyte analyzer (ABL 90 flex, Radiometer; Table 2).

**Table 1.** Composition of culture media

		Culture medium			
Component	Amount	BP	DMEM	NB	NB+
Base medium		BrainPhys	DMEM/F12	Neurobasal-A	Neurobasal-Plus
Penicillin/streptomycin	1:100	+	+	+	+
GlutaMax	1:100	+		+	+
Nonessential amino acids	1:100		+		
B27 supplement	1:50	+		+	
B27 plus supplement	1:50				+
Serum	2%	+	+	+	+
BDNF	10 ng/ml	+	+	+	+
NT3	10 ng/ml	+	+	+	+
Laminin	200 ng/ml	+	+	+	+

**Table 2.** Measured properties of culture media and ACSF

Parameter	Unit	Culture medium				ACSF
		BP	DMEM	NB	NB+	
Osmolarity	mOsm	320	310	270	230	300
K^+^	mM	3.9	4.0	5.0	5.0	2.9
Na^+^	mM	147	151	102	84	148
Ca^2+^	mM	0.86	0.82	1.46	1.46	0.93
Cl^−^	mM	120	126	87	68	126
Glucose	mM	2.3	16.2	23.0	23.8	5.0
Lactate	mM	0.1	0.2	0.0	0.0	0.0

### Rat and mouse primary cortical cultures

Primary cultures from P1 rat and mouse cortex were prepared similar as previously described (Moutin et al., 2020). Animals from both sexes were used. After removal of meninges, the neocortex was dissected and enzymatically digested with Papain (Sigma) in the presence of DNAse (Sigma), followed by mechanical dissociation and centrifugation through a cushion of 4% bovine serum albumin (BSA; Sigma). These steps were completed using Hibernate medium (Invitrogen). Cells were then plated onto poly-D-lysine (Sigma) coated coverslips in 24-well plates. For each coverslip, 25,000 cells were allowed to settle in a 40 µl drop for ∼30 min and then each well was filled with 500 µl growth medium: Neurobasal-A/B27 (1:50, Invitrogen) supplemented with GlutaMax (1:400, Invitrogen), glutamine (0.25–0.5 mM, Sigma), penicillin/streptomycin (1:100, ThermoFisher), and heat-inactivated fetal calf serum (10%, Sigma). Medium was partially exchanged on Day 3 (480 µl) and Day 7 (100 µl) with fresh maintenance medium: BrainPhys (StemCell), B27 (1:50, Invitrogen), GlutaMax (1:400, Invitrogen), and penicillin/streptomycin (1:100, ThermoFisher). Cultures were maintained for up to 3 weeks without any further medium change at 37°C and 5% CO_2_.

### Epifluorescence imaging

**Figure 1.**
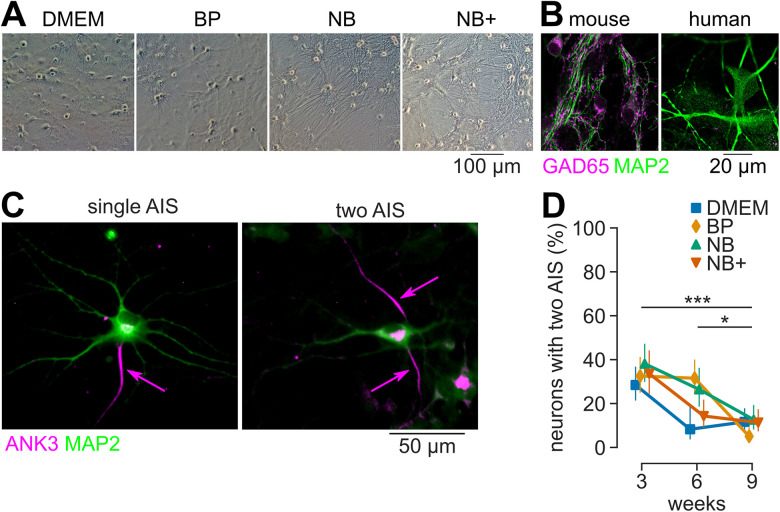
The proportion of cells with multiple axon initial segments decreases to low levels during maturation. ***A***, Bright-field images of cultures of human neurons with mouse astrocytes at 3 weeks. ***B***, Example confocal fluorescence images of neurons stained for GAD65 (magenta) and MAP2 (green) at 6 weeks in human and mouse neuron cultures. ***C***, Example confocal fluorescence images of neurons stained for ANK3 (magenta) and MAP2 (green) at 3 weeks showing neurons with one or two axon initial segments. ***D***, Percentage of neurons with multiple axon initial segments decreases during maturation (*n* = 16–45 neurons per group).

**Figure 2.**
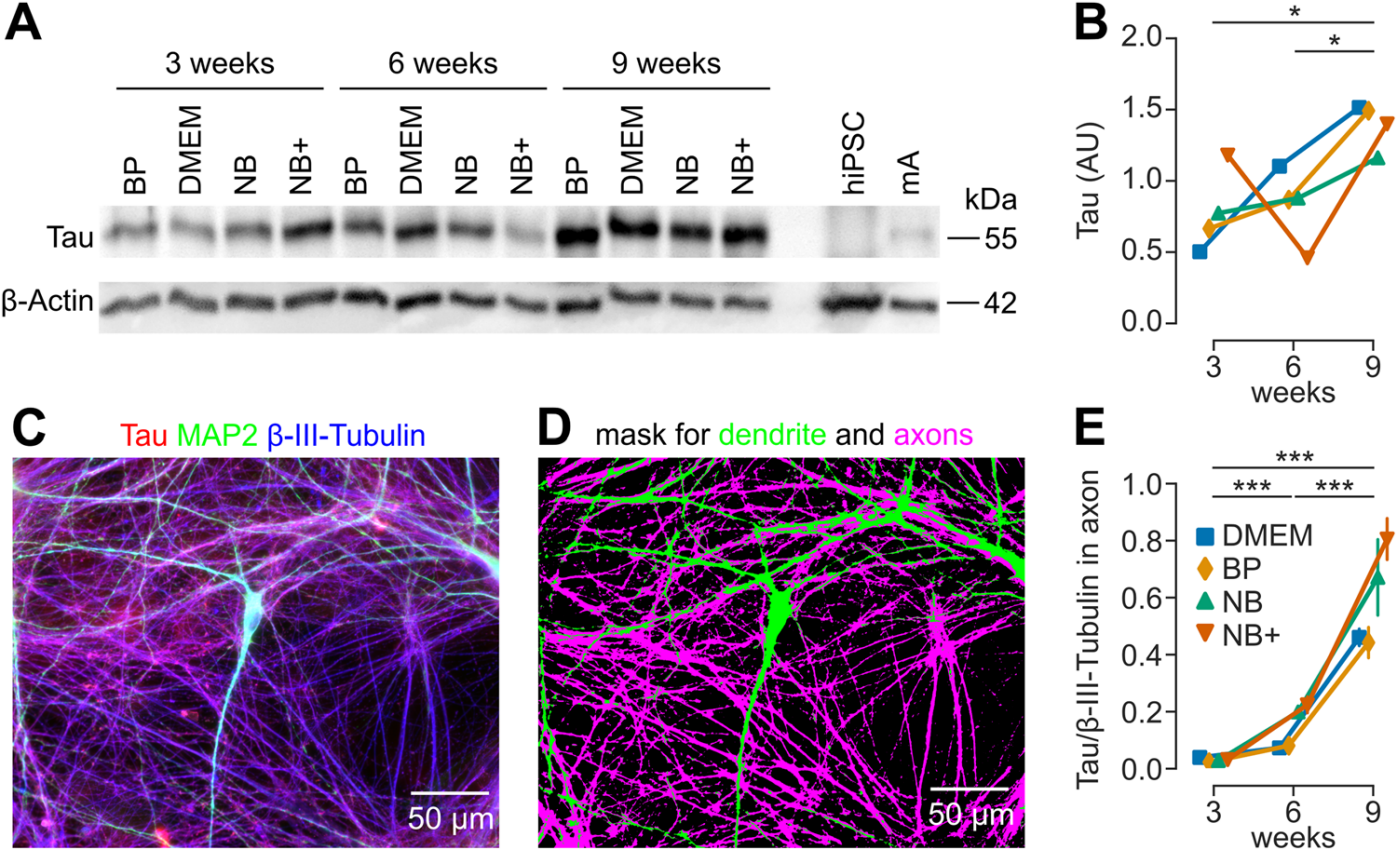
The amount of tau protein in the axon increases during maturation. ***A***, Western blots of total tau in human neurons during maturation in different media. β-Actin was used as a loading control. ***B***, Tau expression increases during maturation of human neurons, quantified from first 12 lanes of the tau Western blot shown in ***A***. ***C***, Example confocal fluorescence images of neurons stained for microtubules (βIII-tubulin, blue) as well as the microtubule-associated proteins tau (red) and MAP2 (green). ***D***, Mask for microtubules in dendrites (MAP2 positive) and axons (MAP2 negative). ***E***, Ratio of tau to tubulin immunofluorescence signals in the axon increases during maturation. Each point represents mean ± standard error from *n* = 5 neurons per age and medium group from 12 independent cultures.

Immunocytochemistry was performed as previously described (Bullmann et al., 2009). Human neurons cocultured with mouse astrocytes were grown on coverslips and fixed for 30 min with 4% paraformaldehyde in PBS, washed twice with PBS + 0.05% sodium azide and stored at 4°C. Fixed cultures were washed with Tris-buffered saline (150 mM NaCl, 50 mM Tris-HCl, pH 7.6) containing 0.05% Triton X-100 (TBS-Tx), unspecific binding was blocked for 30 min with 2% normal donkey serum in TBS-Tx (TBS-Tx-NDS), and primary antibodies were applied in TBS-Tx-NDS overnight at 4°C. The next day, cultures were washed three times with TBS-Tx, and secondary antibodies were applied in TBS-Tx-NDS for 1–2 h at RT. Afterward, cultures were washed three times TBS-Tx and one time with PBS + 0.05% sodium azide and the coverslips were mounted upside down onto microscope slides using Mowiol (ThermoFisher). For axon initial segment counting (Fig. 1*B*), primary antibodies were directed against MAP2 and ANK3 (Table 3), and imaging was performed using ZEISS epifluorescence microscope equipped with 10× air objective. For quantification of tau distribution (Fig. 2*C*), primary antibodies were directed against tau, MAP2, and βIII tubulin (Table 3), and imaging was performed using ZEISS epifluorescence microscope equipped with 40× water immersion objective. Using the MAP2 staining of soma and dendrites as a guidance, images from individual, “nonoverlapping” neurons were taken and analyzed. Note that the image contains not only the axon of this individual neuron but most like the axons of other neurons as well.

**Table 3.** Antibodies, dilutions used for immunocytochemistry, and Western blots

Antibody	Antigen	Dilution	Source	Identifier
Rabbit anti-GAD65/67 antibody	GAD65/67	1:500	Sigma	Catalog #G5163 RRID:AB_477019
Mouse anti-ANK3 antibody, clone N106/36	ANK3	1:1,000	NeuroMAB	Catalog #75_146 RRID:AB_2877524
Polyclonal rabbit anti-MAP2 antibody	MAP2	1:2,000	Millipore	Catalog #AB5622 RRID:AB_91939
Polyclonal rabbit anti-βIII-tubulin antibody	Tubulin	1:2,000	Synaptic Systems	Catalog #302302 RRID:AB_10637424
Polyclonal Rabbit Anti-Homer1 antibody	Homer1	1:500	Synaptic Systems	Catalog #160003 RRID:AB_887730
Monoclonal mouse Anti-Bassoon antibody	Bassoon	1:500	Enzo Life Sciences	Catalog #ADI-VAM-PS003-F RRID:AB_11181058
Monoclonal mouse anti-tau antibody, clone	Tau	1:1,000	DSHB	Catalog #5A6 RRID:AB_528487
Polyclonal Rabbit Anti-Human Tau antibody	Tau	1:4,000	Agilent	Catalog #A0024 RRID:AB_10013724
monoclonal rat anti-beta-actin antibody, clone W16197A	rat	1:1,000	BioLegend	Catalog #664804 RRID:AB_2728496
Anti-Rabbit-HRP	Rabbit IgG	1:20,000	Dianova	Catalog #711-035-152
Anti-mouse-HRP	Mouse and rat IgG	1:20,000	Dianova	Catalog #515-035-151
Donkey-anti-rat- AF647	Rat IgG	1:1,000	Dianova	Catalog #712-605-153
Goat-anti-mouse-StarOrange	Mouse IgG	1:400	Abberior	Catalog #STORANGE-1001-500UG
Goat-anti-rabbit-StarRed	Rabbit IgG	1:400	Abberior	Catalog #STRED-1002-500UG
Goat-anti-guinea pig-AF488	Guinea Pig IgG	1:500	Biozol	Catalog #106-545-003
Donkey-anti-rabbit-AF488	Rabbit IgG	1:500	Biozol	Catalog #711-545-152
Donkey-anti-rabbit-AF647	Rabbit IgG	1:500	Biozol	Catalog #711-605-152
Donkey-anti-mouse-Cy3	Mouse IgG	1:500	Jackson	Catalog #715-165-150

Further information can be found on https://www.antibodyregistry.org/ using the RRID provided for each primary antibody.

### STED imaging

Immunocytochemistry was performed as described for epifluorescence imaging. Briefly, human neurons grown in Neurobasal media for 3, 6, and 9 weeks were fixed and incubated with primary antibodies directed against Bassoon and Homer1 (Table 3) which were then detected using StarRed and StarOrange conjugated secondary antibodies, respectively. Imaging (Fig. 5*A*) was performed using an Expert Line Abberior Confocal and STED microscope equipped with an Olympus (UPlanXApo) 60× oil objective with a numerical aperture of 1.42. The images were obtained with a 10 nm pixel size (2D mode) and sequential line scanning by switching between the needed excitation lasers line-wise during each recording. Synapse labeled both for Bassoon and Homer1 were identified and for each channel a line profile perpendicular to the synaptic cleft was fitted with a Gaussian, and their peak-to-peak distance was measured (Fig. 5*B*). For comparison, this assay was also performed on DIV14 rat cortical neurons.

### Immunoblotting

Immunoblotting was performed as previously described (Bullmann et al., 2009). Briefly, human neurons were maintained in 6-well plates at an initial density of 240,000 neurons/well. After 3, 6 and 9 weeks, cultures were washed using HBSS and cells were extracted directly in Laemmli buffer. Samples were applied to 10% polyacrylamide gel electrophoresis (PAGE) and transferred to PDVS membranes using a tank blotter at 25 V overnight. PDVS membranes were washed with TBS and 0.05% Tween-20 (TBS-Tw), unspecific binding was blocked for 30 min with 2% BSA in TBS-Tw (TBS-Tw-BSA), and primary antibodies were applied in TBS-Tw-BSA overnight at 4°C. The next day, membranes were washed 3× TBS-Tw, and secondary antibodies were applied in TBS-Tw for 1 h at RT. Afterward, membranes were washed 3× TBS-Tx, equilibrated in bioluminescence substrate, and imaged on Fusion FX (Vilber).

### Electron microscopy

Neurons grown for 40 d in Neurobasal medium on coverslips, as done for light microscopy, were fixed with 2% glutaraldehyde in PBS for 1 h, washed in PBS and postfixed in 1% osmium tetroxide in 0.1 M cacodylate buffer. After washing and staining en bloc by 1% aqueous uranyl acetate, cells were dehydrated in methanol gradient series, infiltrated by epoxy resin and polymerized. Coverslips were removed by liquid nitrogen and resin blocks were mounted for ultrathin sectioning. Sixty-nanometer sections were collected on grids and imaged at Zeiss 900 transmission electron microscope.

### Patch-clamp recordings

#### Solutions and recording conditions

Extracellular ACSF contained the following (in mM): 125 NaCl, 3 KCl, 10 glucose, 25 NaH_2_CO_3_, 1.25 Na_2_HPO_4_, 1.1 CaCl_2_, 1 MgCl_2_. The extracellular solution had an osmolarity of 298 mOsm and was continuously equilibrated with 5% CO_2_ and 95% O_2_ to pH 7.35. All recordings were performed at 35–37°C using a Peltier bath perfusion heater (Warner Instruments) with bath temperature sensing. Recording pipettes were filled with potassium gluconate-based (KGlu) internal solution containing the following (in mM): 150 K-gluconate, 3 Mg-ATP, 0.3 Na-GTP, 10 K-HEPES, 10 NaCl, and 0.2 EGTA, pH adjusted to 7.2 by KOH, osmolarity 293 mOsm. For recordings from cultures grown in Neurobasal Plus medium (250–270 mOsm), osmolarities of the internal and external solution were both adjusted to the culture osmolarity by adding *Aqua dest*.

#### Somatic current-clamp recordings

Whole-cell somatic current-clamp recordings were performed with pipettes pulled from borosilicate glass (Science Products) by a DMZ Universal Electrode Puller with filament heating (Zeitz Instruments). Recording pipettes were filled with potassium gluconate-based solution and had a resistance of 4–6.5 MΩ. Membrane potentials were corrected for the 16 mV liquid junction potential calculated from the ion concentrations in the internal and external solutions according the stationary Nernst–Planck equation (Marino et al., 2014) using LJPcalc (https://swharden.com/LJPcalc/).

Pipettes were fixed on a custom-built pipette holder and mounted on a micromanipulator (Kleindiek Nanotechnik). All recordings were sampled at 200 kHz with a 10 kHz low-pass filter (Bessel, 8-pole) using a HEKA EPC10/2 amplifier (HEKA Elektronik) controlled by the PatchMaster software (HEKA Elektronik). Pipette capacitance was compensated with the internal capacitance compensation circuit of the EPC10. The circuit uses a capacitance value that is determined in voltage-clamp mode in the cell-attached configuration and automatically subtracts 0.5 pF to prevent oscillations. The built-in bridge compensation was then adjusted to 100% of the series resistance determined in voltage clamp. For recordings, cultures were mounted on an upright microscope (Nikon FN-1, Nikon Instruments) and visualized by a 60× water-immersion objective (NA 1.0) using difference interference contrast (DIC) optics.

The voltage slope $\left(\dot{V}\right)$ was calculated as the first time derivative of somatic membrane potential (dV / dt). Action potentials were identified as voltage inflections with an upward slope larger than 50 mV/ms. The current threshold (*I*_th_) required for eliciting an action potential was denoted as rheobase. The membrane potential at which the slope reached 25 mV/ms was denoted voltage threshold (*V*_th_). For each action potential, the peak voltage (*V*_max_) and peak slope $\left({\dot{V}}_{\max}\right)$ was measured. The half-width was calculated using the first action potential at rheobase as the action potential duration at the membrane voltage halfway between threshold and peak voltage ((*V*_th _+ *V*_max_) / 2).

#### Postsynaptic voltage-clamp recordings

Extracellular ACSF had the same composition as described above, except using 5 mM glucose and 5 mM sucrose instead of 10 mM glucose. Internal solution was used as described above, except for using 0.05 mM instead of 0.2 mM EGTA. Pipettes had a resistance of 2.5–6.5 MΩ. Excitatory postsynaptic currents (EPSCs) triggered by presynaptic action potentials that were induced by extracellular stimulation were recorded as previously reported (Maximov et al., 2007; Hallermann et al., 2010; Ritzau-Jost et al., 2014). Briefly, somatic recordings were performed as described above from human neurons grown in NB medium. The ACSF contained the selective NMDA receptor antagonist AP-5 (Tocris, 0.05 mM) to isolate AMPA receptor currents recorded in voltage-clamp mode at a holding potential of −70 mV using an EPC-10 (HEKA Elektronik) while correcting for a calculated liquid junction potential of 16 mV. Recordings were sampled at 50 kHz. QX-314 (5 mM) was added to the intracellular solution to block sodium channels to avoid sodium channel activation in incomplete clamped cellular compartments. The access resistance of the somatic recordings was estimated with the built-in whole-cell capacitance compensation circuit of the EPC10 and ranged between 4 and 15 MΩ. The whole-cell capacitance compensation was then deactivated during the recordings.

Stimulation pipettes were also pulled from borosilicate glass but were slightly larger and had an open-tip resistance of 2–3 MΩ when filled with ACSF. Both recording and stimulation pipette were mounted on uMp-3 micromanipulators (SensaPex). After obtaining a whole-cell recording configuration, the stimulation pipette was placed at a distance of 50–200 µm, and 0.1 ms square pulses were delivered while scanning the area until monophasic EPSCs could be reliably recorded at a minimally required stimulation intensity for reliable stimulation of 20–60 V. High-frequency stimulations with 0.1 ms duration and frequency of 10, 50, and 100 Hz were delivered in combination with a consecutive series of six recovery stimuli at an interval of 75, 125, 250, 500, 1,000, and 3,000 ms. The measurements were repeated several times at an interval of 20 s for 10  and 50 Hz and at an interval of 30 s for 100 Hz. All recordings were made at a temperature of 35–37°C.

EPSCs during high-frequency transmission were analyzed as follows: the baseline of each trace was subtracted and an average of all trace from one neuron recording with the same train frequency was calculated and filtered (low-pass 5 kHz Butterworth filter). In these average trains, the stimulation artifacts were removed using blanking window with a total length of 1.8 ms (0.6 ms before the onset of stimulation until 1.2 ms after the end of a given stimulus). For every mean evoked EPSCs, the baseline (0.4–2 ms before onset of the stimulation artifact) and the maximal negative peak (0–4 ms after the onset of the stimulation artifact) was calculated. The paired-pulse ratio (PPR) was calculated as the ratio of the amplitude of the second to the first EPSC during high-frequency transmission. The steady-state amplitude (SS) was calculated as the average of the peaks of the last five EPSCs. The time course of the amplitude of the EPSCs in the recovery phase was fitted using a monoexponential function (constraining for a start at the steady-state value and a recovery to amplitude of the first EPSC) and the subsequent *τ* value was extracted. Average trains with an estimated *τ* value >20 s were excluded for subsequent analysis (*n* = 7 out of 85 average trains in 3 out of 58 neurons). Inclusion of these data led to similar results. The normalized cumulative charge transfer (NCCT) was calculated by integrating the first EPSC of each train for 10 ms from the end of the stimulation artifact (1.2 ms after onset of stimulation) as described before (Uzay et al., 2023). The synaptic jitter was calculated as standard deviation of the trace-to-trace variation in the time of the peak of the EPCS for each stimulation during high-frequency transmission. The decay of the EPSC was fitted with a monoexponential function over a time course over which the signal showed 80 to 20% of the negative peak of a given EPSC. Fits with *R*^2^ < 0.85 were not used (1 out of 15 and 1 out of 13 neurons for 6 and 9 weeks, respectively).

#### Presynaptic recordings

Direct presynaptic current-clamp recordings were performed as previously described (Ritzau-Jost et al., 2021, 2023) from human neurons grown in BrainPhys (9 weeks, *n* = 9) or Neurobasal medium (4 and 10 weeks, *n* = 5 and 4, respectively). Briefly, current-clamp recordings were performed with pipettes pulled from quartz glass (without filament; Heraeus Quarzglas) with a DMZ Universal Electrode Puller with oxygen–hydrogen burner (Zeitz Instruments; Dudel et al., 2000). Pipettes had tip resistances of 6–15 MΩ and were filled with the same potassium gluconate-based internal solution used for somatic recordings but with 100 µM Atto-594 added (ATTO-TEC) for confirmation of the axonal recording (Fig. 7*A*). Voltages were recorded using a MultiClamp 700A patch-clamp amplifier (Molecular Devices), filtered with the internal 10 kHz four-pole Bessel filter, and subsequently digitized (200 kHz) with the HEKA EPC10/2 using PatchMaster software (HEKA Elektronik) or digitized (100 kHz) with the Digidata 1550B using Clampex software 11.2 (Molecular Devices). Pipette capacitance compensation was performed with the hybrid voltage-/current-clamp approach (Ritzau-Jost et al., 2021, 2023), which uses the value of the pipette capacitance determined in the voltage-clamp mode for the pipette capacitance neutralization in the current-clamp mode. To minimize the impact of pipette capacitance on voltage recordings, shortest possible pipettes were fixed on a custom-built pipette holder that enabled a steep pipette immersion angle and mounted on a micromanipulator (Kleindiek Nanotechnik). Low bath perfusion levels were used and silicone grease was applied onto the recording electrode wire to prevent internal solution from being pulled up by adhesive forces and onto the electrode shank under binocular guidance. During recordings, cultures were mounted on an inverted microscope (Nikon TI-U, Nikon Instruments) and visualized by a 100× water-immersion objective (NA 1.1) using DIC optics.

Capacitance measurements were performed as previously described (Ritzau-Jost et al., 2014) at a holding potential of −100 mV and a sine wave stimulation at 8.3 kHz frequency with an amplitude of ±50 mV. The “sine + DC” method of the HEKA EPC10/2 amplifier controlled by PatchMaster software was used (HEKA Elektronik). The intracellular solution contained the following (in mM): 130 CsCl, 0.5 MgCl_2_, 20 TEA-Cl, 20 HEPES, 5 Na_2_ATP, 0.3 NaGTP. The extracellular solution contained the following (in mM): 105 NaCl, 25 NaHCO_3_, 25 glucose, 20 TEA, 5 4-AP, 2.5 KCl, 2 CaCl_2_, 1.25 NaH_2_PO_4_, 1 MgCl_2_, and 0.001 tetrodotoxin (TTX), equilibrated with 95% O_2_ and 5% CO_2_. The sine wave stimulation was interrupted with brief depolarizing pulses to 0 mV for 10 ms duration. The calcium current amplitude was measured in a 2 ms window at the end of the depolarizing pulse (i.e., before the tail current). The change in capacitance was measured in a 100 ms window beginning 50 ms after the end of the depolarizing pulse.

### Experimental design and statistical analysis

Data analysis was performed with custom made scripts in Python (3.11.2) using the package seaborn (0.12.2) for plotting. Statistical testing was performed with the packages scipy (1.10.1) and statsmodels (0.13.5). Line plots show mean and its standard error. For Figure 1*D* (proportion of multiple AIS), the line plot shows mean and its confidence interval calculated by Wilson’s method, and a 68% confidence interval was chosen because this corresponds approximately to the standard error used in the other plots. Box plots (Figs. 5*B*, 7*C*) show median and quantile range.

To test for dependence on culture time and medium, a two-way ANOVA with interactions was performed with post hoc Tukey’s HSD. All-positive data were log transformed which led to normal distributed residuals. We also analyzed our data without transformation using the nonparametric Scheirer–Ray–Hare test (using R version 4.3.1; R Core Team, 2023) which led to the same results. Culture time and medium were treated as ordinal variables.

To test the dependence of the recovery time constant, we performed steady-state depression and paired pulse interval (Fig. 6*D*,*E*,*F*) on culture time and stimulation interval a two-way ANOVA with interactions with post hoc Tukey’s HSD as described above. Culture time and medium were treated as ordinal variables.

If only one factor was considered (Fig. 5*C*) a Kruskal–Wallis *H* test was used. A Mann–Whitney *U* test was used to compare two groups (Fig. 5*C*, pooled human vs rat).

The proportions of neurons possessing multiple AIS (Fig. 1*D*) were compared by the chi-squared test. In all cases, first, dependency on medium was tested for 3, 6, and 9 weeks separately. Second, data were pooled for medium and tested for dependency on culture time. This was followed by multiple pairwise tests (3 vs 3, 3 vs 6, 6 vs 9 weeks) with *p* value correction according to the Benjamin–Hochberg procedure.

Western blots (Fig. 2*B*) were quantified using FIJI/ImageJ by measuring the optical density of the band and subtracting the optical density of the background. For each combination of the culture media (DMEM, BP, NB, NB+ medium) and culture age (3, 6, 9 weeks), we obtained one measurement (total of *n* = 12 measurements). We then performed two-way-ANOVA without interactions (degrees of freedom, df = 6).

For presynaptic recordings, data for 9- and 10-week-old neurons were pooled after no significant differences were observed for action potential overshoot (*V*_max_; Mann–Whitney *U* test; *p* = 0.11) as well as half-width (Mann–Whitney *U* test; *p* = 0.43).

## Results

### The proportion of cells with multiple axon initial segments decreases to low levels during maturation

To identify suitable conditions for the structural and functional maturation of human iPSC-derived neurons, we studied neurons cultured with mouse astrocytes for 3, 6, and 9 weeks in four different commonly used media based on Dulbecco’s modified Eagle’s medium (DMEM), BrainPhys (BP), Neurobasal (NB), and Neurobasal Plus (NB+). First, we confirmed that these culture do not contain GABAergic neurons by immunolabeling for GAD65/67, which is present in the presynaptic compartment of GABAergic neurons only. Indeed, we hardly detected any GAD65/67 immunolabeling in the human cultures compared with mouse neocortical neuron cultures (Fig. 1*B*). Second, we checked the specification of the axon initial segment and the maturation of the axon proper. Approximately 30% of human neurons generated by NGN2-overexpression previously had multiple axons when grown in island (autaptic) cultures after 4 to 6 weeks in vitro (Meijer et al., 2019; Rhee et al., 2019). We therefore investigated the age and medium dependence of the occurrence of multiple axons in human iPSC-derived neurons in dissociated (mass) cultures forming 2D networks. Similar to autaptic neurons, the proportion of neurons with multiple axons in our cultures was ∼30% after 3 weeks in culture and across the four tested media (Fig. 1*C*,*D*). However, the proportion of neurons with multiple axons decreased during maturation (chi-square test: *p* < 0.001). Pairwise chi-square tests with Benjamini–Hochberg correction revealed a significant difference between 3 and 9 weeks (*p* < 0.001) and 6 and 9 weeks (*p* = 0.02), but not between 3 and 6 weeks (Tukey’s HSD: *p* = 0.23). At 9 weeks, ∼10% of the neurons possessed two instead of one AIS. There was no statistically significant dependence on the growth medium (chi-square test: *p* = 0.65). These data indicate a medium-independent structural maturation of the human iPSC-derived neurons up to 9 weeks in culture.

### The amount of tau protein in the axon increases during maturation

A hallmark of axonal maturation is the increasing axonal content of tau protein throughout development in vitro (Kempf et al., 1996) and in vivo (Fiock et al., 2020). We therefore quantified total tau protein levels by immunoblotting (Fig. 2*A*) for three different time points (3, 6, or 9 weeks of culture age) and four different media. Two-way ANOVA for tau levels showed a significant increase during development (Fig. 2*B*; *p* = 0.047) but showed no significant influence of culture medium (*p* = 0.976). Next, we investigated the molecular maturation of axons focusing on the localization of tau protein. To this end, we imaged cultures immunolabeled for tau protein, pan-neuronally expressed βIII-tubulin, and dendritically expressed MAP2 by confocal microscopy (Fig. 2*C*). Images were then segmented and axons were identified based on the presence of βIII-tubulin and the absence of MAP2 (Fig. 2*D*). Human neurons showed extensive axonal arborizations and large dendritic trees. Axonal tau immunofluorescence was then compared with axonal βIII-tubulin immunofluorescence. During development, the ratio of tau bound to axonal microtubules strongly increased (Fig. 2*E*; ANOVA: *p* < 0.001). Post hoc test revealed a significant difference between 3, 6, and 9 weeks (Tukey’s HSD: *p* < 0.001) but not between different media (Tukey’s HSD: *p* > 0.46). These data indicate a continuous maturation throughout development from 3 to 9 weeks.

### Passive properties indicate continuous cell growth and a stable resting membrane potential after 6 weeks

**Figure 3.**
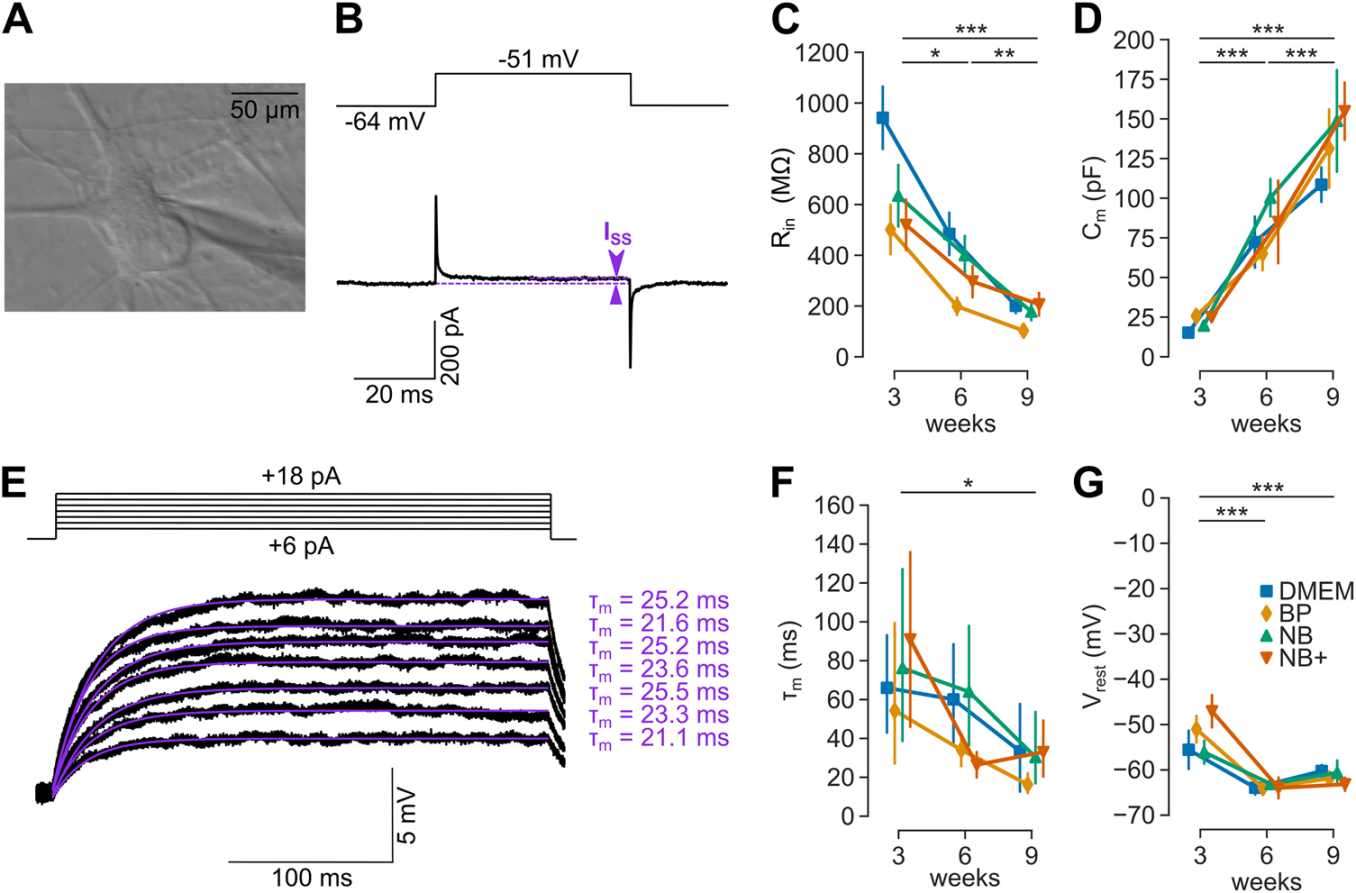
Passive properties indicate continuous cell growth and a stable resting membrane potential after 6 weeks. ***A***, Example DIC image of a whole-cell patch-clamp recording from a 6-week-old neuron. ***B***, Example of a square-pulse stimulus (with 100 ms duration and 5 mV amplitude). The input resistance (*R*_in_) was calculated from the steady-state current (*I*_ss_). ***C***, The input resistance *R*_in_. ***D***, The cell capacitance *C_m_*. ***E***, Example of membrane voltage responses to a series of current injections. The membrane time constant (*τ_m_*) was estimated by fitting a monoexponential function to each voltage response. ***F***, The membrane time constant (*τ_m_*). ***G***, The resting membrane potential *V*_rest_. Recorded neurons per group: *n* = 8–26, except (***C***) with *n* = 6–19 and (***F***) with *n* = 3–12.

After having found structural and molecular axonal development in human iPSC-derived neurons, we set out to determine changes in the passive membrane properties and action potential firing as a measure of functional maturation. We recorded the currents elicited by depolarizing voltage steps of 5 mV in somatic whole-cell recordings in four different media and at three developmental ages (3, 6, and 9 weeks; Fig. 3*A*,*B*). From the passive transients, we calculated the input resistance (*R*_in_) and the cell capacitance (*C_m_*, a measure of total neuronal surface area). During development, *R*_in_ decreased (Fig. 3*C*; ANOVA: *p* < 0.001) irrespective of the medium, except for a difference between BP and DMEM (Tukey’s HSD: *p*_adj _= 0.022). Since we did not determine the seal resistance systematically (knowing only that it was >1 GΩ), the input resistance of the neurons could have been underestimated. In contrast to the decrease of *R*_in_, *C*_m_ increased ∼4-fold (Fig. 3*D*; ANOVA: *p* < 0.001) suggesting continuous cell growth throughout maturation from 3 to 9 weeks. In addition, we measured the membrane time constant (*τ_m_*) by fitting the membrane voltage responses to a series of current injections (Fig. 3*E*). During development, *τ_m_* decreased (Fig. 3*F*; ANOVA: *p* < 0.001) irrespective of the medium (ANOVA: *p* = 0.401). Finally, we measured the resting membrane potential (*V*_rest_), which decreased during development (Fig. 3*G*; ANOVA: *p* < 0.001). Interestingly, *V*_rest_ stabilized after 6 weeks (Tukey’s HSD for 6 vs 9 week: *p*_adj _= 0.55) at approximately −64 mV across all four tested media (ANOVA: *p* = 0.45). These data indicate that despite the continuous growth from 3 to 9 weeks, the functional maturity assessed by the resting membrane potential reached a plateau after 6 weeks.

### Somatic action potential duration decreased and excitability increased during maturation

**Figure 4.**
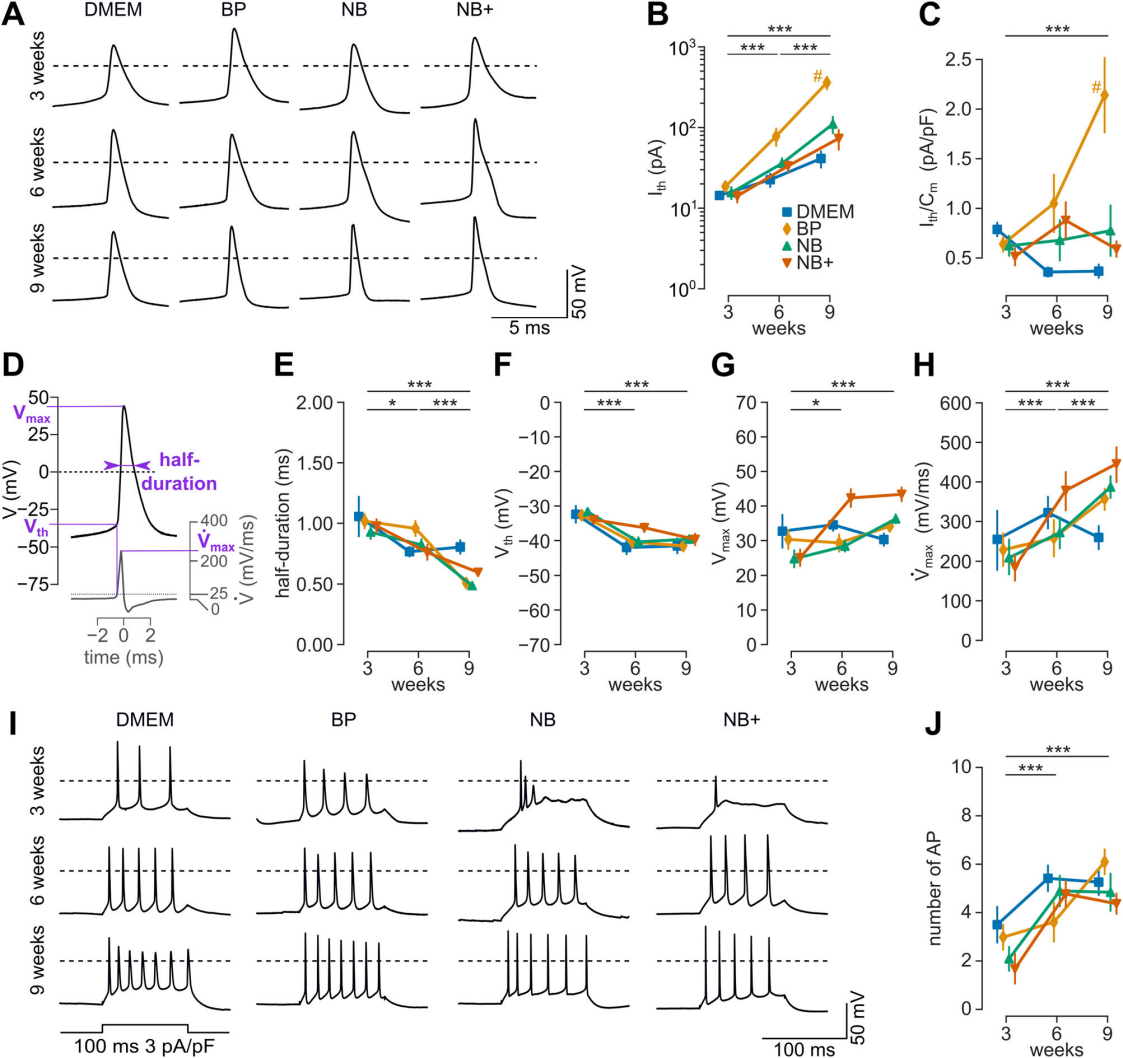
Somatic action potential duration decreases and excitability increases during maturation. ***A***, Examples of somatic action potentials recorded in ACSF after development in different culture media (dashed lines indicate 0 mV potentials). ***B***, The current threshold *I*_th_. ***C***, The current threshold divided by the cell capacitance *C*_m_ (current density threshold). ***D***, Example showing the parameters used to describe an action potential, including half-duration, threshold voltage *V*_th_, and peak voltage *V*_max_. ***E***, Action potential half-duration. ***F***, Action potential threshold voltage *V*_th_. ***G***, Action potential peak voltage *V*_max_. ***H***, Action potential maximum slope ${\dot{V}}_{\max}$. ***I***, Examples of action potentials elicited by square pulse stimuli (with 100 ms duration and a current density of 3 pA/pF) in ACSF after development in different culture media (dashed lines indicate 0 mV potentials). ***J***, The number of action potentials in response to a current injection (current density of 3 pA/pF). Recorded neurons per group: *n* = 8–31, except (***I***) with *n* = 7–14.

As a more relevant parameter of functional maturity, we analyzed action potential firing. We recorded action potentials in the different media and developmental stages (Fig. 4*A*). To quantify the basic excitability, we measured the rheobase as the current threshold (*I*_th_) required to elicit an action potential during 50 ms of current injection. *I*_th_ increased developmentally in all media (Fig. 4*B*; ANOVA: *p* < 0.001), with similar ∼8-fold increases in *I*_th_ in neurons grown in all media except from BP which showed a ∼12-fold *I*_th_ increase (Tukey’s HSD: *p*_adj _= 0.0004, 0.011, 0.076 for BP vs DMEM, NB, NB+, respectively). When normalized by the neuronal surface (*C_m_*; Fig. 3*C*), threshold currents appeared fairly stable during maturation except from BP (Fig. 4*C*; ANOVA: *p* = 0.013; Tukey’s HSD: *p*_adj _< 0.002 for BP vs other media). Next, the impact of cultivation age and media on action potential shape were determined (Fig. 4*D*, also see Materials and Methods). The half-duration of action potentials measured at near-physiological recording temperature (34°C) decreased developmentally (Fig. 4*E*; ANOVA: *p* < 0.001). The voltage threshold (*V*_th_) of action potentials decreased by ∼10 mV from 3 to 9 weeks in culture across all media (Fig. 4*F*; ANOVA: *p* < 0.001; ∼8 mV for NB medium) and the peak voltage (*V*_max_) slightly increased (Fig. 4*G*; ANOVA: *p* < 0.001; e.g., ∼12 mV for NB medium). Finally, the maximum upward voltage slope $\left({\dot{V}}_{\max}\right)$ was measured (Fig. 4*H*) which showed a developmental increase from ∼200 to ∼400 mV/ms (ANOVA; *p* < 0.001; Tukey’s HSD; *p*_adj _= 0.001 between all age groups). Furthermore, it depended on culture medium (ANOVA; *p* = 0.024) and at 9 weeks ${\dot{V}}_{\max}$ was lower in DMEM (∼250 mV/ms) when compared with NB medium (Tukey’s HSD; *p*_adj _= 0.008; ∼380 mV/ms) or NB+ medium (*p*_adj _= 0.001; ∼450 mV/ms).

Finally, we determined developmental changes in repetitive firing of action potentials in all four media evoked by incremental current injections (Fig. 4*I*). To account for the variability in cell size under the experimental conditions, the current injection amplitude was normalized to the cell capacitance. The number of action potentials fired at current injections of 3 pA/pF significantly increased from 3 to 6 weeks (two-way ANOVA; *p* < 0.001; Tukey’s HSD; *p*_adj _= 0.001) but not afterward (*p*_adj _= 0.693; Fig. 4*J*). The number of action potentials obtained after 6 to 9 weeks approximately corresponds to a frequency of 50 Hz. These data indicate that the capability for high-frequency action potential firing reached maturity upon 6 weeks, independent of the culture medium.

### Human-induced neurons develop synapses with narrow apposition of pre- and postsynaptic scaffolding proteins

**Figure 5.**
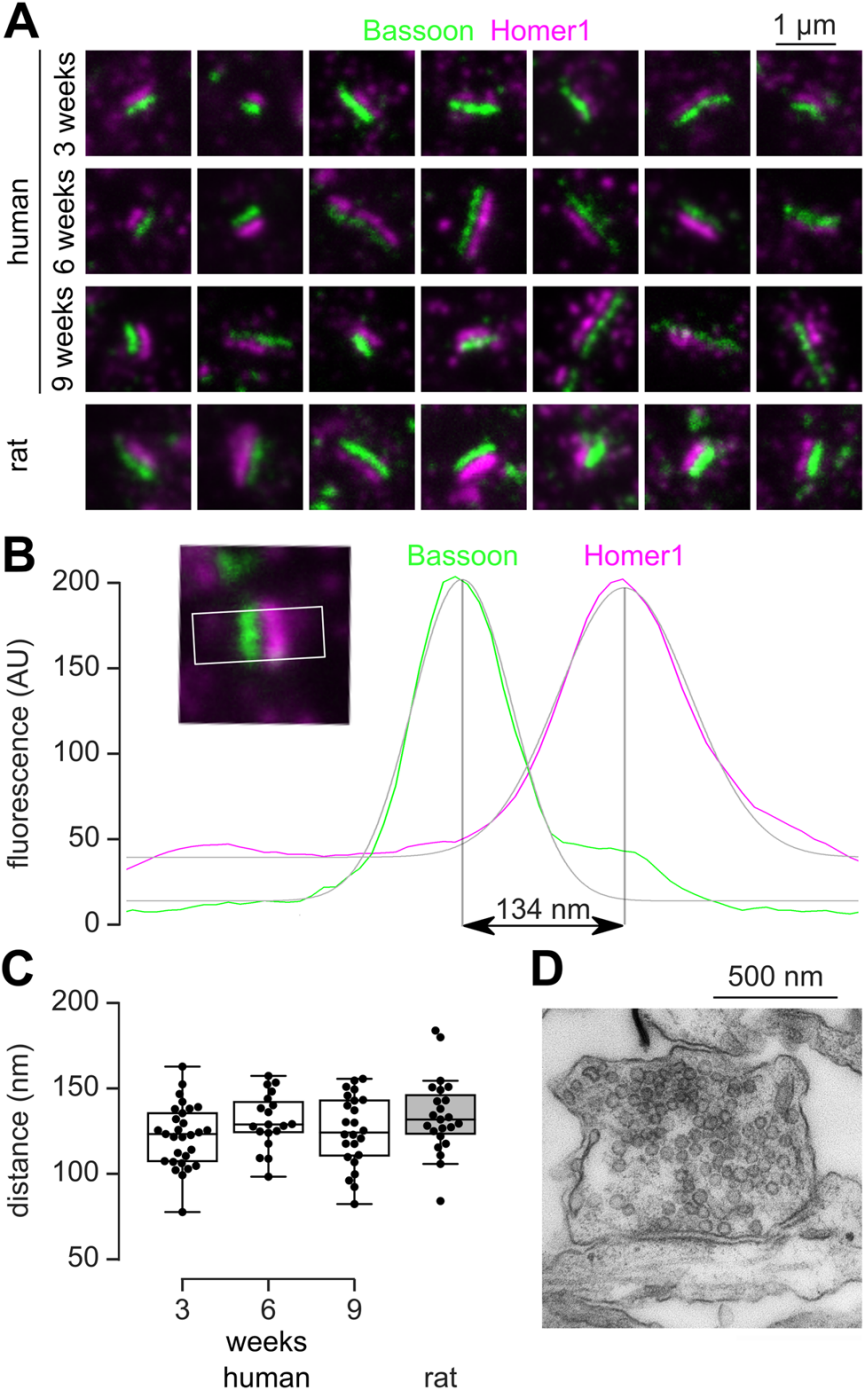
Human-induced neurons develop synapses with narrow apposition of pre- and postsynaptic scaffolding proteins. ***A***, Examples of STED images showing the ultrastructure of synapses with distinct pre- and postsynaptic labeling of Bassoon (green) and Homer1 (magenta) at 3, 6, and 9 weeks. ***B***, For measurement of peak-to-peak distance between presynaptic Bassoon and postsynaptic Homer1, synaptic contacts were aligned (small insert) and line profiles (white box) were fitted to Gaussians (gray line). ***C***, During development a nonsignificant change in the distance of presynaptic Bassoon and postsynaptic Homer1 was observed. For comparison, measurements are shown for rat cortical neurons at DIV14. Number of synapses per group: *n* = 29, 19, 24, 23. ***D***, Example electron microscopic image of a synaptic contact between human neurons cultured for 7 weeks.

Structural synapse development was determined by super-resolution stimulated emission depletion (STED) microscopy and electron microscopy. Double immunolabeling of the presynaptic marker Bassoon and the postsynaptic marker for glutamatergic synapses Homer1 revealed synaptic contacts as early as 3 weeks in any of the four culture media (Fig. 5*A*). For neurons grown in Neurobasal medium, the distance between the maximum intensities of Bassoon and Homer1 was measured for synapses oriented perpendicular to the optical plane (Fig. 5*B*). This distance did not change during development (Fig. 5*C*; Kruskal–Wallis *H* test: *p* = 0.252) and was similar to synapses in rat cortical neuron cultures (Mann–Whitney *U* test: *p* = 0.125). This distance (∼130 nm) was only slightly smaller than the Bassoon-Homer1 distances measured from synapses in the accessory olfactory bulb (∼155 nm) and cortex of adult mice (∼150 nm) using STORM microscopy (Dani et al., 2010). Furthermore, electron microscopic images (three individual coverslips; *n* = 50 boutons observed) revealed presynaptic vesicles, a synaptic cleft, and a postsynaptic density, reminiscent of mature vertebrate synapses (representative example shown in Fig. 5*D*). These data indicate that induced human neurons form proper glutamatergic synapses with characteristic molecular and ultrastructural features.

### High-frequency transmission with synchronous release up to 100 Hz

**Figure 6.**
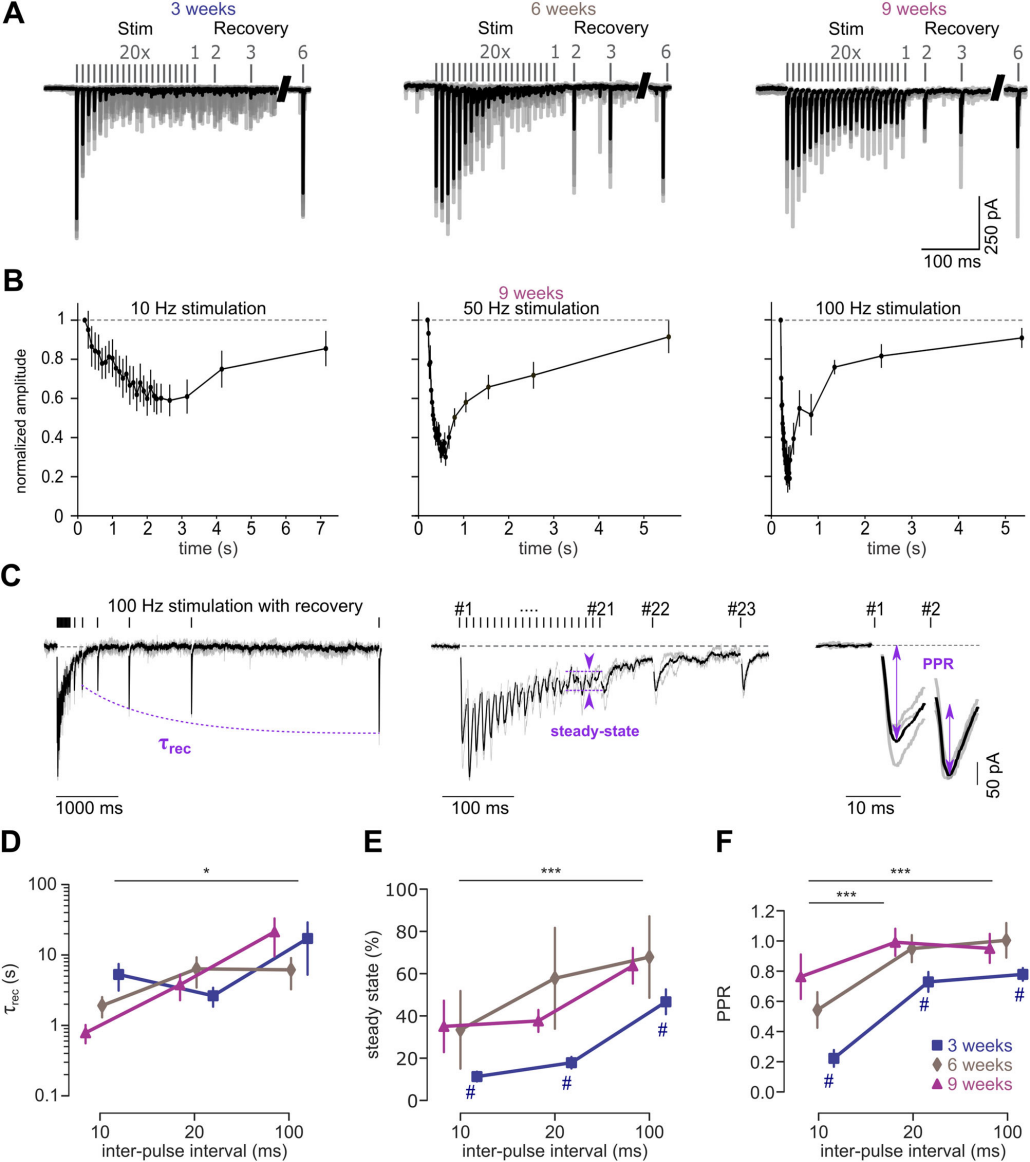
High-frequency transmission with synchronous release up to 100 Hz. ***A***, Example traces of evoked EPSCs elicited by high-frequency stimulation at 50 Hz and 6 pulses of recovery (with exponentially increasing time difference) at different developmental stages (3, 6, and 9 weeks). The average (black) and individual consecutive traces of a recording from an individual cell (gray) are superimposed (stimulation artifacts removed). ***B***, Average amplitudes normalized to first EPSC of a series of EPSCs with stimulation at 10, 50, and 100 Hz at the age of 9 weeks. ***C***, Example traces of evoked EPSCs in a 6-week-old neuron elicited by high-frequency stimulation at 100 Hz and 6 pulses of recovery at different time scales illustrating the analysis of recovery from depression, the steady-state amplitude, and the PPR (stimulation artifacts removed). ***D***, Steady-state amplitude (normalized amplitude of the last 5 EPSCs during high-frequency transmission). ***E***, PPR calculated from the amplitude of first two EPSCs in a trace. ***F***, Time constant of a monoexponential fit (*τ*_rec_) of the EPSC amplitudes during the recovery. Recordings without recovery due to strong facilitation were excluded from the analysis of the recovery kinetics. Number of neurons per group are 20, 29, and 21 for 3, 6, and 9 weeks, respectively. Except for ***B*** with 11, 13, and 6 neurons for 10, 50, and 100 Hz, respectively.

To analyze the function of these synapses, we measured short-term plasticity during high-frequency synaptic transmission and the recovery from synaptic depression at 3, 6, and 9 weeks in vitro. To this end, we recorded EPSCs evoked by extracellular stimulation with a second pipette at a frequency of 10, 50, and 100 Hz at near-physiological recording temperature (35–37°C). Evoked EPSCs could be reliably measured already after 3 weeks in vitro (Fig. 6*A*). Furthermore, there was a substantial developmental change over the course of several weeks in vitro. We analyzed the time constant of recovery from short-term depression, the steady-state EPSC amplitude of the last five EPSCs, and the PPR of the first two EPSCs as illustrated in Figure 6*C* for EPSCs elicited by a 100 Hz stimulation. The recovery from depression accelerated with higher stimulation frequencies (two-way ANOVA; *p* = 0.013), but there was no significant dependency on age (two-way ANOVA; *p* = 0.09; Fig. 6*D*; *n* = 78 trains from *n* = 55 neurons; each train being the average of 1 to 19 traces from one neuron with the same train frequency). The amount of short-term depression assessed by the normalized steady-state amplitude increased with the stimulation frequency in all age groups (two-way ANOVA; *p* < 0.001; *n* = 85 trains from 58 neurons; Fig. 6*E*). However, short-term depression decreased during maturation from 3 to 9 weeks (two-way ANOVA; *p* < 0.001). This decrease was significant between 3 and 6 weeks but not between 6 and 9 weeks (post hoc Tukey’s HSD; *p* = 0.018 and *p* = 0.38, respectively; *n* = 26, 29, and 30 trains for 3, 6, and 9 weeks, respectively; Fig. 6*E*). Consistently, the PPR increased with frequency and decreased during maturation (two-way ANOVA; *p* < 0.001 for both). This decrease was significant between 3 and 6 weeks but not between 6 and 9 weeks (Tukey’s HSD; *p* < 0.001 and *p* = 0.62, respectively; Fig. 6*F*). At 50 Hz stimulation, the proportion of facilitating synapses (i.e., PPR > 1) was 11% (1 out of 9 neurons), 27% (4 out of 15 neurons), and 54% (7 out of 13 neurons) for 3, 6, and 9 weeks in culture, respectively. These data indicate an increase in the fidelity of synaptic transmission during maturation from 3 to 9 weeks, reaching reliable synaptic transmission at frequencies up to 100 Hz at 6 and 9 weeks.

### Temporal precision of synaptic transmission increases during development

**Figure 7.**
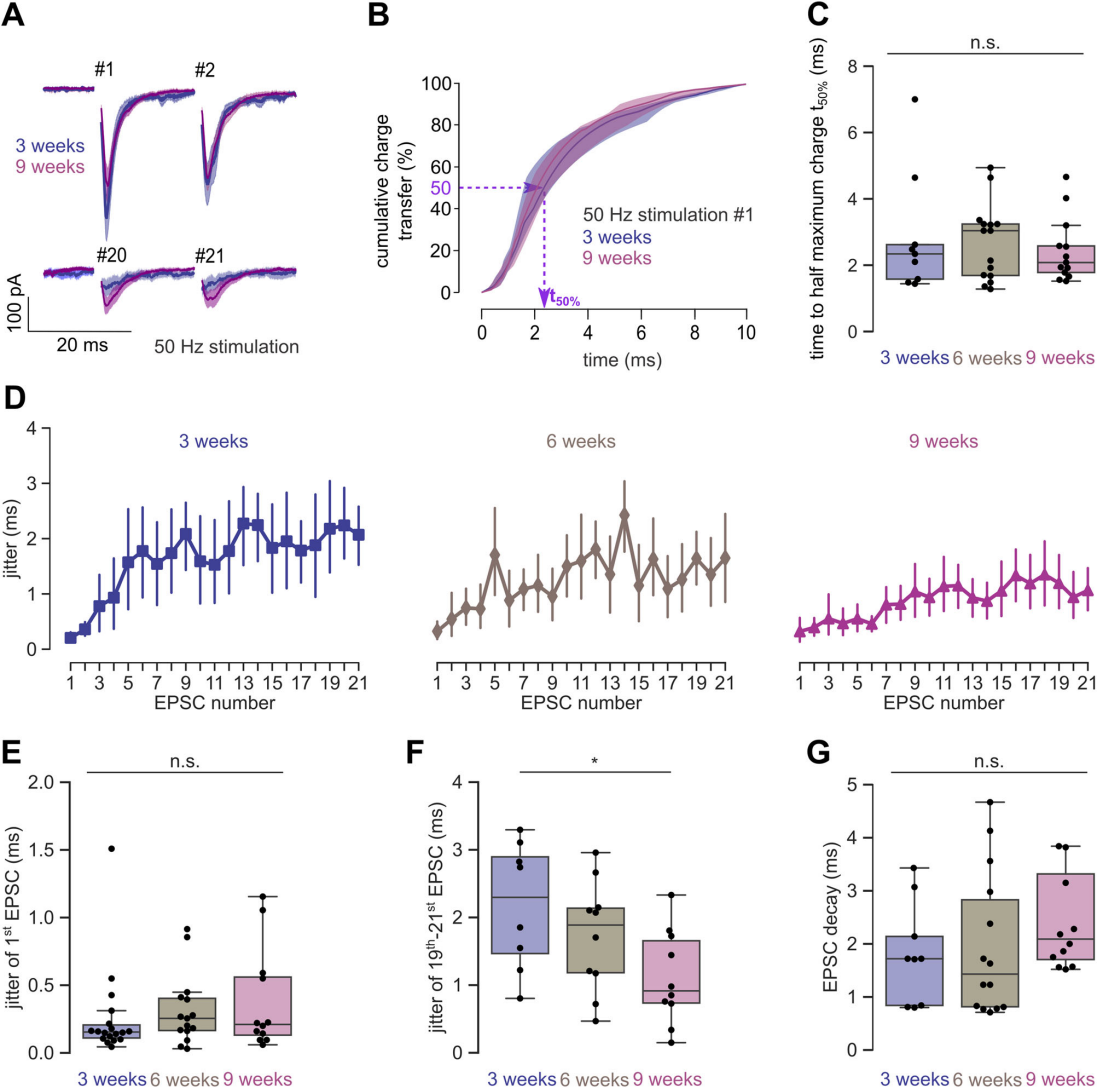
Temporal precision of synaptic transmission increases during development. ***A***, Grand averages of the first and last EPSCs of a 50 Hz stimulation at 3 and 9 weeks shown as mean ± standard error. ***B***, The NCCT for the first EPSC of a 50 Hz stimulation (median and interquartile range, for *n* = 9 and 13 neurons for 3 and 9 weeks, respectively). ***C***, The time to the half of the normalized maximal charge transfer for the first EPSC during 50 Hz stimulation (*n* = 9, 15, and 13 neurons for 3, 6, and 9 weeks, respectively). ***D***, Average jitter of the time of the peak of the EPSC during 50 Hz stimulation (*n* = 8, 10, and 10 neurons for 3, 6, and 9 weeks, respectively; only including neurons with at least 3 traces to calculate the jitter). ***E***, Jitter of the first EPSC (*n* = 18, 15, and 12 neurons for 3, 6, and 9 weeks, respectively; using the first EPSC from the 20, 50, and 100 Hz stimulation; only including neurons with at least 3 traces to calculate the jitter). ***F***, Average jitter of the last three EPSCs during the 50 Hz stimulation as shown in panel ***D*** (*n* = 8, 10, and 10 neurons for 3, 6, and 9 weeks, respectively). ***G***, Decay time constant for the first EPSC during 50 Hz stimulation (*n* = 9, 14, and 12 neurons for 3, 6, and 9 weeks, respectively; only including monoexponential fits with *R*^2^ > 0.85).

To analyze the temporal precision of synaptic transmission, we first analyzed the synchronicity of synaptic transmission. In contrast to a study that reported an age-dependent desynchronization of postsynaptic currents in iPSC-derived human neurons (Uzay et al., 2023), we found little change in the synchronicity between 3 and 9 weeks. If anything, the grand average of all trains showed a higher peak and less asynchronous release at 9 weeks, particularly during the last two EPSCs (Fig. 7*A*, #20 and #21). Moreover, the NCCT for first 10 ms starting from the peak of the first EPSC during the high-frequency stimulations (Fig. 7*B*) did not show a prominent right shift as observed by Uzay et al. (2023). Correspondingly, the time to half-maximal charge transfer (*t*_50%_) did not change significantly during development (*p* = 0.664; Kruskal–Wallis test; *n* = 9, 15, and 13 neurons for 3, 6, and 9 weeks, respectively; Fig. 7*C*).

As another measure of temporal precision of synaptic transmission, we calculated the standard deviation of the trace-to-trace variation (i.e., the jitter) in the time of the peak of the EPCSs during high-frequency transmission for each neuron and averaged these standard deviations across all neurons. Repetitive stimulation led to a pronounced increase of the EPSC jitter, but with notable differences at 3, 6, and 9 weeks (Fig. 7*D*). The initial EPSC jitter did not change significantly during development (*p* = 0.309; Kruskal–Wallis test; *n* = 45 neurons; using the first EPSC of either stimulation frequency; Fig. 7*E*). In contrast, the jitter of the last EPCSs during high-frequency transmission decrease significantly during development (*p* = 0.050; Kruskal–Wallis test; *n* = 28 cells; using the 19th to 21st EPCSs from 50 Hz stimulations; *p* = 0.012 between 3 and 9 weeks; Mann–Whitney test; Fig. 7*F*). Finally, we analyzed the EPSC decay time constant, which depends on several parameters but also on the amount of asynchronous release. However, also the decay time constant did not change during development (*p* = 0.181; Kruskal–Wallis; *n* = 9, 14, and 12 neurons for 3, 6, and 9 weeks, respectively; Fig. 7*G*). These data did not reveal signs of desynchronization of release during maturation and indicate an increased temporal precision of synaptic transmission during maturation.

### Presynaptic action potentials are large and rapid

**Figure 8.**
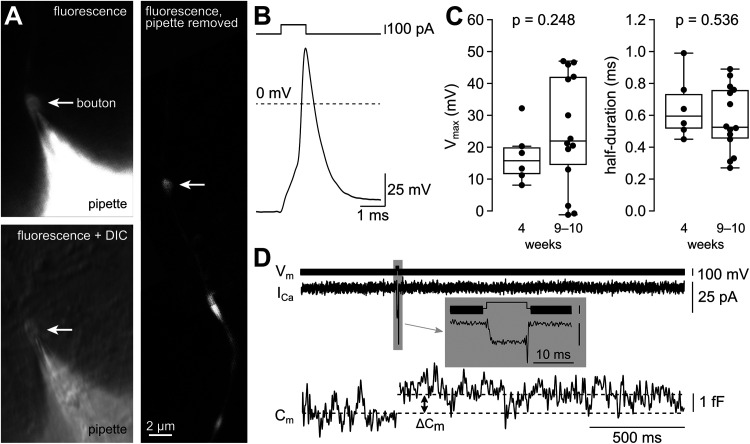
Presynaptic axon potentials are large and rapid. ***A***, Example image of a whole-cell patch-clamp recording from a bouton of an 9-week-old iPSC-derived neuron showing the fluorescence of the ATTO 488 dye in the pipette solution (top left), the superposition of the fluorescence and the DIC image (bottom left), and the florescence due to filling of the axon with the dye after withdrawal of the patch pipette (right). ***B***, Example of a presynaptic action potential at 9 weeks. ***C***, Peak amplitude and half-duration of the presynaptic action potentials (*n* = 6 and14 bouton recordings at 4 and 9–10 weeks, respectively). ***D***, Example of presynaptic capacitance recording showing voltage command (*V_m_*), the pharmacologically isolated calcium current (*I*_Ca_), and the membrane capacitance (*C_m_*). The elicited increase in *C*_m_ is indicated (Δ*C_m_*). Gray marked inset shows a magnification of *V_m_* and *I*_Ca_ (calibration, 100 mV and 25 pA).

The high fidelity and precision of synaptic transmission indicates that induced human neurons can serve as a model to study the mechanisms of synaptic transmission. To analyze the presynaptic mechanisms in more detail, we aimed to establish presynaptic whole-cell patch-clamp recordings in human neurons, similar to the method previously described for cultured mouse neurons (Ritzau-Jost et al., 2021, 2023). Specifically, we visually identified presynaptic boutons in DIC microscopy. Moreover, in selected experiments, we used costaining with FM 1-43 Dye (Gaffield and Betz, 2007) to confirm that the visually identified structures indeed represent presynaptic boutons capable of vesicle cycling. In each recording, the recorded structure was filled with the fluorescent dye ATTO 488 contained in the intracellular pipette solution. The diameter of the boutons was ∼1 µm and additional en passant boutons along the axon were visible (Fig. 8*A*).

To determine central parameters controlling the precision of synaptic transmission, we measured presynaptic action potentials with current-clamp recordings. As previously shown (Ritzau-Jost et al., 2021), the pipette capacitance limits the temporal resolution and the amplitude of the measured action potentials. We therefore lowered the height of the solution in the bath chamber to a minimum and used short quartz glass pipettes (Dudel et al., 2000) and pipettes minimally filled with intracellular solution (Ritzau-Jost et al., 2021, 2023). Brief current injections (1 ms) were applied to elicit presynaptic action potentials (Fig. 8*B*). Quantification of the maximum peak potential (overshoot) and the action potential half-duration reveal large and rapid action potentials (Fig. 8*C*) at near-physiological recording temperature (35–37°C). At 4 weeks, the peak potential was +16 [12, 20] mV and the half-duration was 0.60 [0.52, 0.73] ms (median [interquartile range]; *n* = 6). At 9–10 weeks, the peak potential was +22 [15, 42] mV and the half-duration was 0.53 [0.46, 0.76] ms (median [interquartile range]; *n* = 14). There were no significant differences for amplitude (Mann–Whitney *U* test; *p* = 0.27) and half-duration (Mann–Whitney *U* test; *p* = 0.56). The amplitude and half-duration were similar to corresponding recordings of presynaptic action potentials from conventional small boutons in cultured neocortical neurons of mice (Ritzau-Jost et al., 2021).

To test whether we recorded from functional presynaptic nerve terminals, we performed capacitance measurements (Fig. 8*D*), which allow direct monitoring of exo- and endocytosis. Depolarizations to 0 mV for 10 ms elicited calcium currents of 16.40 [9.70, 30.15] pA amplitude (median [interquartile range]; *n* = 6) and an increase in the membrane capacitance of 0.83 [0.24, 1.00] fF (median [interquartile range]; *n* = 6). These properties are similar to previous corresponding recordings from small conventional boutons of vertebrate neurons (Kawaguchi and Sakaba, 2017). Therefore, the structures we recorded from represent functional presynaptic terminals. Because action potential recordings and capacitance measurements cannot be made simultaneously from the same boutons, we cannot completely exclude the possibility that some of the action potentials were recorded from neuronal structures that are not functional presynaptic terminals. Taken together, however, these data suggest that the presynaptic action potentials of human glutamatergic neurons are large and fast, with properties that are similar to those that have previously been measured in vertebrate neurons.

## Discussion

Here we investigated the mechanisms of synaptic transmission in human neurons derived from iPSCs by transient expression of Ngn2. We show (1) that the maturation of morphological, molecular, and electrophysiological properties of these neurons is similar in different commonly used culture media, (2) that the fidelity and the temporal precision during high-frequency synaptic transmission increases during maturation, (3) that 9-week-old neurons exhibit reliable synchronous synaptic transmission up to a frequency of 100 Hz, and (4) that the presynaptic action potentials have a large amplitude and short duration with no change during maturation from 4 to 9–10 weeks. Our results establish Ngn2-induced iPSC-derived neurons as a model for high-resolution structure–function analyses of synaptic transmission and uncover fundamental parameters of glutamatergic signaling between human neurons.

### Synchronous high-frequency transmission

The here-used induced human neurons exhibit structural (one AIS; Fig. 1), molecular (tau in axon; Fig. 2), and functional properties (resting membrane potential and action potential firing; Figs. 3, 4) similar to mature murine neurons. Furthermore, synapses showed characteristic structural signs of maturation (Fig. 5) and surprisingly reliable transmission up to 100 Hz (Figs. 6, 7). The fidelity and synchronicity of neurotransmitter release seemed higher compared with autaptic human neurons (Fenske et al., 2019; Meijer et al., 2019; Rhee et al., 2019), but this might be due to the physiological recording temperatures used here (Pyott and Rosenmund, 2002). In a recent study analyzing 3- to 7-week-old cultures of Ngn2-induced neurons, the absence of inhibitory neurons was found to cause endoplasmic reticulum stress and augmented asynchronous release in neurons older than 5 weeks in vitro (Uzay et al., 2023). However, we did not observe such a decrease in synchronicity of release during maturation from 3 to 9 weeks in vitro (Fig. 7*C*). Instead, we observed a developmental increase in the temporal precision of synaptic transmission during high-frequency synaptic transmission (Fig. 7*D–F*). There are several factors that could have contributed to this different finding: first, although we use the same Ngn2 overexpression method to differentiate glutamatergic neurons from iPSCs, our iPSC line is different from the one used in the previous study. Second, we use 2% fetal calf serum throughout the culture, which might increase viability of the neuron cultures and prevent the premature aging phenotype. Independent of the varying onset of aging processes in this in vitro model, the here-observed surprisingly synchronous release up to a transmission frequency of 100 Hz seems consistent with the emerging view that human neurons are particularly tuned to high-frequency signaling (Testa-Silva et al., 2014; Goriounova et al., 2018; Galakhova et al., 2022).

### Presynaptic patch-clamp recordings in human neurons

The reliability and high-frequency bandwidth of synaptic transmission indicates that transiently expressing Ngn2 in iPSC-derived neurons leads to rather mature glutamatergic transmission. To decipher the mechanisms of synchronous release during high-frequency transmission in humans, we established direct presynaptic patch-clamp recordings from boutons of 4- to 10-week-old human neurons. Presynaptic recordings represent a valuable tool to analyze presynaptic function with an exceptionally high temporal resolution (Neher, 1998; Vandael et al., 2021). The technique has been applied to large synapses in the vertebrate brain, such as the calyx of Held in the brainstem and mossy fiber boutons in the hippocampus and cerebellum (reviewed by von Gersdorff and Borst, 2002; Bischofberger et al., 2006; Delvendahl and Hallermann, 2016). Here, we extended the applicability of this technique from vertebrate to human synapses.

### Human presynaptic action potentials

Presynaptic patch-clamp recordings allowed measuring large and rapid presynaptic action potentials at the calyx of Held and the hippocampal and cerebellar mossy fiber bouton (overshoot potential ∼30 to 50 mV, half duration ∼0.1 to 0.4 ms at 35–37°C; Geiger and Jonas, 2000; Taschenberger and Von Gersdorff, 2000; Ishikawa et al., 2003; Alle et al., 2011; Ritzau-Jost et al., 2014). However, there is still some uncertainty regarding the properties of presynaptic action potentials at small conventional synapses in the vertebrate brain. For example, genetically encoded voltage sensors indicate that the amplitudes of presynaptic action potentials in small conventional boutons of cultured hippocampal neurons is only 70% of the amplitude at the soma (0 mV overshoot; Hoppa et al., 2014). Furthermore, the extracellular voltage decreased while propagating along the axon (Radivojevic et al., 2017), suggesting a decreased action potential amplitude in more distal axons. It was also reported that the duration slightly decreased in distal compared with proximal axons (Gonzalez Sabater et al., 2021). In addition to these optical and extracellular techniques, high-resolution current-clamp recordings from small conventional boutons of cultured neurons of mice and pyramidal neurons in acute brain slices of mice were performed indicating large and rapid presynaptic action potentials (overshoot approximately +30 to +50 mV, half duration ∼0.3 to 0.4 ms at 35–37°C; Oláh et al., 2021; Ritzau-Jost et al., 2021). We here establish this technique for small conventional boutons of human iPSC-derived neurons and measured comparable parameters (Fig. 8*C*). Thus, despite fundamental differences between human and rodent neurons, our data indicated that glutamatergic synaptic transmission is mediated by large and rapid presynaptic action potentials in both rodents and humans. The rapid and large presynaptic action potentials enable fast activation and deactivation of presynaptic calcium channels (Sabatini and Regehr, 1996; Borst and Sakmann, 1998; Bischofberger et al., 2002), which contributes to here-observed reliable and synchronous neurotransmitter release during high-frequency transmission in human neurons. The presynaptic action potentials exhibited no change during development (Fig. 8*C*). Therefore, the increase in the fidelity (Fig. 6) and temporal precision of synaptic transmission (Fig. 7) during development is most likely caused by other factors including, e.g., the developmental acceleration of vesicle recruitment (Taschenberger and Von Gersdorff, 2000; Yamashita et al., 2010) or decrease of the calcium channel-to-vesicle coupling distance (Wang et al., 2008; Bornschein et al., 2019; Chen et al., 2024).

### In vitro maturation of human neurons

The commonly used DMEM-based media have not been developed for culturing neurons. Therefore, the family of Neurobasal media (Brewer et al., 1993) and most notably the BrainPhys medium (Bardy et al., 2015) have been promoted as more suitable for neuronal cultures (Satir et al., 2020; Zabolocki et al., 2020). The latter has an ionic composition close to the ACSF widely used for electrophysiological recordings. We systematically tested different cell culture media but detected only slight differences in the morphological, molecular, and electrophysiological properties, except for a significantly stronger increase in threshold current in BrainPhys medium. This was surprising because there are notable differences in the ionic composition between different media (Tables 1 and 2). Previous reports with chemically differentiated neurons showed that BrainPhys accelerates maturation compared to a mixture of DMEM and Neurobasal-A medium (Satir et al., 2020). However, in autaptic cultures of human neurons induced by Ngn2 expression, BrainPhys seemed to have a negative effect on astrocytes and did not enhance synaptic transmission (Rhee et al., 2019).

To accelerate the maturation of stem cells to neurons, we adopted transient Ngn2 overexpression (Zhang et al., 2013). Ngn2 encodes a neural-specific basic helix–loop–helix (bHLH) transcription factor that is a strong driver of proneural genes (Hulme et al., 2022) and was shown to generate excitatory glutamatergic neurons from murine embryonic stem cells (Thoma et al., 2012). The formation of supernumerous axons was observed in autaptic cultures (Meijer et al., 2019; Rhee et al., 2019) but primary vertebrate neurons can also have multiple AISs (Guo et al., 2017; Huang and Rasband, 2018). It seems plausible to assume that the Ngn2-induced forced maturation causes the supernumerous axons, but Ngn2 expression does not affect the proportion of neurons with multiple axons (Rhee et al., 2019). Independent of the mechanistic reasons for supernumerous axons, our data indicate that within 9 weeks of maturation 90% of all induced neurons harbor only a single axon.

### Perspectives

Our results demonstrate that Ngn2-induced iPSC-derived neurons represent a convenient and simple-to-establish model to study synaptic function at high spatial and temporal resolution in human neurons. The here-established presynaptic patch-clamp recordings have the potential to uncover physiological and pathophysiological mechanisms of neuronal communication in humans (Patzke et al., 2019; Verhage and Sørensen, 2020). Indeed, several iPSC cell lines harboring mutations in the tau gene associated with familial frontotemporal dementia and parkinsonism (FTDP-tau) are already available (Karch et al., 2019), and more will be provided by the recently announced *iPSC Neurodegenerative Disease Initiative* (Ramos et al., 2021).

## References

[B2] Alle H, Kubota H, Geiger JRP (2011) Sparse but highly efficient Kv3 outpace BKca channels in action potential repolarization at hippocampal mossy fiber boutons. J Neurosci 31:8001–8012. 10.1523/JNEUROSCI.0972-11.2011 21632922 PMC6622861

[B3] Bardy C, et al. (2015) Neuronal medium that supports basic synaptic functions and activity of human neurons in vitro. Proc Natl Acad Sci U S A 112:E2725–E2734. 10.1073/pnas.1504393112 25870293 PMC4443325

[B4] Beaulieu-Laroche L, Toloza EHS, van der Goes MS, Lafourcade M, Barnagian D, Williams ZM, Eskandar EN, Frosch MP, Cash SS, Harnett MT (2018) Enhanced dendritic compartmentalization in human cortical neurons. Cell 175:643–651.e14. 10.1016/j.cell.2018.08.045 30340039 PMC6197488

[B5] Bischofberger J, Engel D, Frotscher M, Jonas P (2006) Timing and efficacy of transmitter release at mossy fiber synapses in the hippocampal network. Pflugers Arch 453:361–372. 10.1007/s00424-006-0093-2 16802161

[B6] Bischofberger J, Geiger JRP, Jonas P (2002) Timing and efficacy of Ca2+ channel activation in hippocampal mossy fiber boutons. J Neurosci 22:10593–10602. 10.1523/JNEUROSCI.22-24-10593.2002 12486151 PMC6758411

[B7] Bornschein G, Eilers J, Schmidt H (2019) Neocortical high probability release sites are formed by distinct Ca2+ channel-to-release sensor topographies during development. Cell Rep 28:1410–1418.e4. 10.1016/j.celrep.2019.07.008 31390556

[B8] Borst JGG, Sakmann B (1998) Calcium current during a single action potential in a large presynaptic terminal of the rat brainstem. J Physiol 506:143–157. 10.1111/j.1469-7793.1998.143bx.x 9481678 PMC2230710

[B9] Brewer GJ, Torricelli JR, Evege EK, Price PJ (1993) Optimized survival of hippocampal neurons in B27-supplemented Neurobasal, a new serum-free medium combination. J Neurosci Res 35:567–576. 10.1002/jnr.490350513 8377226

[B10] Bullmann T, Holzer M, Mori H, Arendt T (2009) Pattern of tau isoforms expression during development in vivo. Int J Dev Neurosci 27:591–597. 10.1016/j.ijdevneu.2009.06.001 19540327

[B11] Chen JJ, Kaufmann WA, Chen C, Arai I, Kim O, Shigemoto R, Jonas P (2024) Developmental transformation of Ca2+ channel-vesicle nanotopography at a central GABAergic synapse. Neuron 112:755–771. 10.1016/j.neuron.2023.12.002 38215739

[B12] Dani A, Huang B, Bergan J, Dulac C, Zhuang X (2010) Superresolution imaging of chemical synapses in the brain. Neuron 68:843–856. 10.1016/j.neuron.2010.11.021 21144999 PMC3057101

[B13] Debanne D, Campanac E, Bialowas A, Carlier E, Alcaraz G (2011) Axon physiology. Physiol Rev 91:555–602. 10.1152/physrev.00048.2009 21527732

[B14] Delvendahl I, Hallermann S (2016) The cerebellar mossy fiber synapse as a model for high-frequency transmission in the mammalian CNS. Trends Neurosci 39:722–737. 10.1016/j.tins.2016.09.006 27771145

[B15] Dudel J, Hallermann S, Heckmann M (2000) Quartz glass pipette puller operating with a regulated oxy-hydrogen burner. Pflugers Arch 441:175–180. 10.1007/s004240000407 11211101

[B16] Eyal G, et al. (2016) Unique membrane properties and enhanced signal processing in human neocortical neurons. Elife 5:1–18. 10.7554/eLife.16553 27710767 PMC5100995

[B17] Fenske P, Grauel MK, Brockmann MM, Dorrn AL, Trimbuch T, Rosenmund C (2019) Autaptic cultures of human induced neurons as a versatile platform for studying synaptic function and neuronal morphology. Sci Rep 9:1–13. 10.1038/s41598-019-41259-1 30894602 PMC6427022

[B18] Fiock KL, Smalley ME, Crary JF, Pasca AM, Hefti MM (2020) Increased tau expression correlates with neuronal maturation in the developing human cerebral cortex. eNeuro 7:ENEURO.0058-20.2020. 10.1523/ENEURO.0058-20.2020 32393582 PMC7262004

[B19] Fişek M, Häusser M (2020) Are human dendrites different? Trends Cogn Sci 24:411–412. 10.1016/j.tics.2020.03.002 32392467 PMC7903140

[B20] Gaffield MA, Betz WJ (2007) Imaging synaptic vesicle exocytosis and endocytosis with FM dyes. Nat Protoc 1:2916–2921. 10.1038/nprot.2006.476 17406552

[B21] Galakhova AA, Hunt S, Wilbers R, Heyer DB, de Kock CPJ, Mansvelder HD, Goriounova NA (2022) Evolution of cortical neurons supporting human cognition. Trends Cogn Sci 26:909–922. 10.1016/j.tics.2022.08.012 36117080 PMC9561064

[B22] Geiger JRP, Jonas P (2000) Dynamic control of presynaptic Ca2+ inflow by fast-inactivating K+ channels in hippocampal mossy fiber boutons. Neuron 28:927–939. 10.1016/S0896-6273(00)00164-1 11163277

[B23] Ghatak S, Dolatabadi N, Trudler D, Zhang X, Wu Y, Mohata M, Ambasudhan R, Talantova M, Lipton SA (2019) Mechanisms of hyperexcitability in Alzheimer’s disease hiPSC-derived neurons and cerebral organoids vs. Isogenic control. Elife 8:1–22. 10.7554/eLife.50333 31782729 PMC6905854

[B24] Gidon A, Zolnik TA, Fidzinski P, Bolduan F, Papoutsi A, Poirazi P, Holtkamp M, Vida I, Larkum ME (2020) Dendritic action potentials and computation in human layer 2/3 cortical neurons. Science 367:83–87. 10.1126/science.aax6239 31896716

[B25] Gonzalez Sabater V, Rigby M, Burrone J (2021) Voltage-gated potassium channels ensure action potential shape fidelity in distal axons. J Neurosci 41:5372–5385. 10.1523/JNEUROSCI.2765-20.2021 34001627 PMC8221596

[B26] Goriounova NA, et al. (2018) Large and fast human pyramidal neurons associate with intelligence. Elife 7:1–21. 10.7554/eLife.41714 30561325 PMC6363383

[B27] Guo Y, Liu Z, Chen YK, Chai Z, Zhou C, Zhang Y (2017) Neurons with multiple axons have functional axon initial segments. Neurosci Bull 33:641–652. 10.1007/s12264-017-0169-3 28828584 PMC5725383

[B28] Hallermann S, Fejtova A, Schmidt H, Weyhersmüller A, Silver RA, Gundelfinger ED, Eilers J (2010) Bassoon speeds vesicle reloading at a central excitatory synapse. Neuron 68:710–723. 10.1016/j.neuron.2010.10.026 21092860 PMC3004039

[B29] Hawkins RD, Kandel ER, Siegelbaum SA (1993) Learning to modulate transmitter release: themes and variations in synaptic plasticity. Annu Rev Neurosci 16:625–665. 10.1146/annurev.ne.16.030193.003205 8096376

[B30] Hodge RD, et al. (2019) Conserved cell types with divergent features in human versus mouse cortex. Nature 573:61–68. 10.1038/s41586-019-1506-7 31435019 PMC6919571

[B31] Hoppa MB, Gouzer G, Armbruster M, Ryan TA (2014) Control and plasticity of the presynaptic action potential waveform at small CNS nerve terminals. Neuron 84:778–789. 10.1016/j.neuron.2014.09.038 25447742 PMC4283217

[B32] Huang CYM, Rasband MN (2018) Axon initial segments: structure, function, and disease. Ann N Y Acad Sci 1420:46–61. 10.1111/nyas.13718 29749636 PMC5992072

[B33] Hulme AJ, Maksour S, St-Clair Glover M, Miellet S, Dottori M (2022) Making neurons, made easy: the use of Neurogenin-2 in neuronal differentiation. Stem Cell Rep 17:14–34. 10.1016/j.stemcr.2021.11.015 34971564 PMC8758946

[B34] Ishikawa T, Nakamura Y, Saitoh N, Li WB, Iwasaki S, Takahashi T (2003) Distinct roles of Kv1 and Kv3 potassium channels at the calyx of Held presynaptic terminal. J Neurosci 23:10445–10453. 10.1523/JNEUROSCI.23-32-10445.2003 14614103 PMC6741004

[B35] Ishizuka Y, Bramham CR (2020) A simple DMSO-based method for cryopreservation of primary hippocampal and cortical neurons. J Neurosci Methods 333:108578. 10.1016/j.jneumeth.2019.108578 31899209

[B36] Kaeser PS, Regehr WG (2014) Molecular mechanisms for synchronous, asynchronous, and spontaneous neurotransmitter release. Annu Rev Physiol 76:333–363. 10.1146/annurev-physiol-021113-170338 24274737 PMC4503208

[B37] Karch CM, et al. (2019) A comprehensive resource for induced pluripotent stem cells from patients with primary tauopathies. Stem Cell Rep 13:939–955. 10.1016/j.stemcr.2019.09.006 31631020 PMC6895712

[B38] Kawaguchi S, Sakaba T (2017) Fast Ca2+ buffer-dependent reliable but plastic transmission at small CNS synapses revealed by direct bouton recording. Cell Rep 21:3338–3345. 10.1016/j.celrep.2017.11.072 29262314

[B39] Kempf M, Clement A, Faissner A, Lee G, Brandt R (1996) Tau binds to the distal axon early in development of polarity in a microtubule- and microfilament-dependent manner. J Neurosci 16:5583–5592. 10.1523/JNEUROSCI.16-18-05583.1996 8795614 PMC6578978

[B40] Lin HC, et al. (2021) NGN2 induces diverse neuron types from human pluripotency. Stem Cell Rep 16:2118–2127. 10.1016/j.stemcr.2021.07.006 34358451 PMC8452516

[B41] Marino M, Misuri L, Brogioli D (2014) A new open source software for the calculation of the liquid junction potential between two solutions according to the stationary Nernst-Planck equation. ArXiv 1403.3640 Available at: http://arxiv.org/abs/1403.3640

[B42] Maximov A, Pang ZP, Tervo DGR, Südhof TC (2007) Monitoring synaptic transmission in primary neuronal cultures using local extracellular stimulation. J Neurosci Methods 161:75–87. 10.1016/j.jneumeth.2006.10.009 17118459

[B43] Meijer M, et al. (2019) A single-cell model for synaptic transmission and plasticity in human iPSC-derived neurons. Cell Rep 27:2199–2211. 10.1016/j.celrep.2019.04.058 31091456

[B44] Miller G (2010) Is pharma running out of brainy ideas? Science 329:502–504. 10.1126/science.329.5991.502 20671165

[B45] Molnár G, Rózsa M, Baka J, Holderith N, Barzó P, Nusser Z, Tamás G (2016) Human pyramidal to interneuron synapses are mediated by multi-vesicular release and multiple docked vesicles. Elife 5:1–12. 10.7554/eLife.18167 27536876 PMC4999310

[B46] Monday HR, Younts TJ, Castillo PE (2018) Long-term plasticity of neurotransmitter release: emerging mechanisms and contributions to brain function and disease. Annu Rev Neurosci 41:299–322. 10.1146/annurev-neuro-080317-062155 29709205 PMC6238218

[B47] Moutin E, Hemonnot AL, Seube V, Linck N, Rassendren F, Perroy J, Compan V (2020) Procedures for culturing and genetically manipulating murine hippocampal postnatal neurons. Front Synaptic Neurosci 12:1–16. 10.3389/fnsyn.2020.00019 32425766 PMC7204911

[B48] Nakamura M, et al. (2019) Pathological progression induced by the frontotemporal dementia-associated R406W Tau mutation in patient-derived iPSCs. Stem Cell Rep 13:684–699. 10.1016/j.stemcr.2019.08.011 31543469 PMC6829766

[B49] Neher E (1998) Vesicle pools and Ca2+ microdomains: new tools for understanding their roles in neurotransmitter release. Neuron 20:389–399. 10.1016/S0896-6273(00)80983-6 9539117

[B50] Neher E, Sakaba T (2008) Multiple roles of calcium ions in the regulation of neurotransmitter release. Neuron 59:861–872. 10.1016/j.neuron.2008.08.019 18817727

[B51] Oláh VJ, Tarcsay G, Brunner J (2021) Small size of recorded neuronal structures confines the accuracy in direct axonal voltage measurements. eNeuro 8:ENEURO.0059-21.2021. 10.1523/ENEURO.0059-21.2021 34257077 PMC8342265

[B52] Patzke C, Brockmann MM, Dai J, Gan KJ, Grauel MK, Fenske P, Liu Y, Acuna C, Rosenmund C, Südhof TC (2019) Neuromodulator signaling bidirectionally controls vesicle numbers in human synapses. Cell 179:498–513.e22. 10.1016/j.cell.2019.09.011 31585084 PMC7159982

[B53] Peng Y, et al. (2024) Directed and acyclic synaptic connectivity in the human layer 2-3 cortical microcircuit. Science 384:338–343. 10.1126/science.adg8828 38635709

[B54] Peng Y, Mittermaier FX, Planert H, Schneider UC, Alle H, Geiger JRP (2019) High-throughput microcircuit analysis of individual human brains through next-generation multineuron patch-clamp. Elife 8:e48178. 10.7554/eLife.48178 31742558 PMC6894931

[B55] Pyott SJ, Rosenmund C (2002) The effects of temperature on vesicular supply and release in autaptic cultures of rat and mouse hippocampal neurons. J Physiol 539:523–535. 10.1113/jphysiol.2001.013277 11882684 PMC2290147

[B56] Radivojevic M, Franke F, Altermatt M, Müller J, Hierlemann A, Bakkum DJ (2017) Tracking individual action potentials throughout mammalian axonal arbors. Elife 6:1–23. 10.7554/eLife.30198 28990925 PMC5633342

[B57] Ramos DM, Skarnes WC, Singleton AB, Cookson MR, Ward ME (2021) Tackling neurodegenerative diseases with genomic engineering: a new stem cell initiative from the NIH. Neuron 109:1080–1083. 10.1016/j.neuron.2021.03.022 33831364 PMC8985232

[B1] R Core Team (2023) R: a language and environment for statistical computing. Vienna, Austria: R Foundation for Statistical Computing.

[B58] Rhee HJ, et al. (2019) An autaptic culture system for standardized analyses of iPSC-derived human neurons. Cell Rep 27:2212–2228.e7. 10.1016/j.celrep.2019.04.059 31091457

[B59] Ritzau-Jost A, Delvendahl I, Rings A, Byczkowicz N, Harada H, Shigemoto R, Hirrlinger J, Eilers J, Hallermann S (2014) Ultrafast action potentials mediate kilohertz signaling at a central synapse. Neuron 84:152–163. 10.1016/j.neuron.2014.08.036 25220814

[B60] Ritzau-Jost A, Nerlich J, Kaas T, Krueger M, Tsintsadze T, Eilers J, Barbour B, Smith SM, Hallermann S (2023) Direct whole-cell patch-clamp recordings from small boutons in rodent primary neocortical neuron cultures. STAR Protoc 4:102168. 10.1016/j.xpro.2023.102168 36920913 PMC10026040

[B61] Ritzau-Jost A, Tsintsadze T, Krueger M, Ader J, Bechmann I, Eilers J, Barbour B, Smith SM, Hallermann S (2021) Large, stable spikes exhibit differential broadening in excitatory and inhibitory neocortical boutons. Cell Rep 34:108612. 10.1016/j.celrep.2020.108612 33440142 PMC7809622

[B62] Rizalar FS, et al. (2023) Phosphatidylinositol 3,5-bisphosphate facilitates axonal vesicle transport and presynapse assembly. Science 382:223–230. 10.1126/science.adg1075 37824668 PMC10938084

[B63] Russell WMS, Burch RL (1959) The principles of humane experimental technique. Methuen.

[B64] Sabatini BL, Regehr WG (1996) Timing of neurotransmission at fast synapses in the mammalian brain. Nature 384:170–172. 10.1038/384170a0 8906792

[B65] Satir TM, Nazir FH, Vizlin-Hodzic D, Hardselius E, Blennow K, Wray S, Zetterberg H, Agholme L, Bergström P (2020) Accelerated neuronal and synaptic maturation by BrainPhys medium increases Aβ secretion and alters Aβ peptide ratios from iPSC-derived cortical neurons. Sci Rep 10:1–17. 10.1038/s41598-019-56847-4 31953468 PMC6969066

[B66] Sohn PD, et al. (2019) Pathogenic tau impairs axon initial segment plasticity and excitability homeostasis. Neuron 104:458–470.e5. 10.1016/j.neuron.2019.08.008 31542321 PMC6880876

[B67] Südhof TC (2012) The presynaptic active zone. Neuron 75:11–25. 10.1016/j.neuron.2012.06.012 22794257 PMC3743085

[B68] Südhof TC (2013) Neurotransmitter release: the last millisecond in the life of a synaptic vesicle. Neuron 80:675–690. 10.1016/j.neuron.2013.10.022 24183019 PMC3866025

[B69] Taschenberger H, Von Gersdorff H (2000) Fine-tuning an auditory synapse for speed and fidelity: developmental changes in presynaptic waveform, EPSC kinetics, and synaptic plasticity. J Neurosci 20:9162–9173. 10.1523/JNEUROSCI.20-24-09162.2000 11124994 PMC6773022

[B70] Testa-Silva G, Verhoog MB, Linaro D, de Kock CPJ, Baayen JC, Meredith RM, De Zeeuw CI, Giugliano M, Mansvelder HD (2014) High bandwidth synaptic communication and frequency tracking in human neocortex. PLoS Biol 12:e1002007. 10.1371/journal.pbio.1002007 25422947 PMC4244038

[B71] Thoma EC, Wischmeyer E, Offen N, Maurus K, Sirén AL, Schartl M, Wagner TU (2012) Ectopic expression of neurogenin 2 alone is sufficient to induce differentiation of embryonic stem cells into mature neurons. PLoS ONE 7:e38651. 10.1371/journal.pone.0038651 22719915 PMC3374837

[B72] Ting JT, et al. (2018) A robust ex vivo experimental platform for molecular-genetic dissection of adult human neocortical cell types and circuits. Sci Rep 8:1–13. 10.1038/s41598-018-26803-9 29849137 PMC5976666

[B73] Tracy TE, et al. (2022) Tau interactome maps synaptic and mitochondrial processes associated with neurodegeneration. Cell 185:712–728. 10.1016/j.cell.2021.12.041 35063084 PMC8857049

[B74] Uzay B, Houcek A, Ma ZZ, Konradi C, Monteggia LM, Kavalali ET (2023) Neurotransmitter release progressively desynchronizes in induced human neurons during synapse maturation and aging. Cell Rep 42:112042. 10.1016/j.celrep.2023.112042 36701235 PMC10366341

[B75] Vandael D, Okamoto Y, Borges-Merjane C, Vargas-Barroso V, Suter BA, Jonas P (2021) Subcellular patch-clamp techniques for single-bouton stimulation and simultaneous pre- and postsynaptic recording at cortical synapses. Nat Protoc 16:2947–2967. 10.1038/s41596-021-00526-0 33990799

[B76] Verhage M, Sørensen JB (2020) SNAREopathies: diversity in mechanisms and symptoms. Neuron 107:22–37. 10.1016/j.neuron.2020.05.036 32559416

[B77] von Gersdorff H, Borst JGG (2002) Short-term plasticity at the calyx of held. Nat Rev Neurosci 3:53–64. 10.1038/nrn705 11823805

[B78] Wang LY, Neher E, Taschenberger H (2008) Synaptic vesicles in mature calyx of Held synapses sense higher nanodomain calcium concentrations during action potential-evoked glutamate release. J Neurosci 28:14450–14458. 10.1523/JNEUROSCI.4245-08.2008 19118179 PMC6671236

[B79] Werner C, Sauer M, Geis C (2021) Super-resolving microscopy in neuroscience. Chem Rev 121:11971–12015. 10.1021/acs.chemrev.0c01174 33752325

[B80] Yamashita T, Eguchi K, Saitoh N, von Gersdorff H, Takahashi T (2010) Developmental shift to a mechanism of synaptic vesicle endocytosis requiring nanodomain Ca2+. Nat Neurosci 13:838–844. 10.1038/nn.2576 20562869 PMC3677945

[B81] Zabolocki M, et al. (2020) Brainphys neuronal medium optimized for imaging and optogenetics in vitro. Nat Commun 11:5550. 10.1038/s41467-020-19275-x 33144563 PMC7642238

[B82] Zhang Y, et al. (2013) Rapid single-step induction of functional neurons from human pluripotent stem cells. Neuron 78:785–798. 10.1016/j.neuron.2013.05.029 23764284 PMC3751803

[B83] Zhou B, Lu JG, Siddu A, Wernig M, Südhof TC (2022) Synaptogenic effect of APP -Swedish mutation in familial Alzheimer’s disease. Sci Transl Med 9380:eabn9380. 10.1126/scitranslmed.abn9380 36260691 PMC9894682

